# Evaluating high-fidelity CRISPR-Cas nucleases in nucleosomal contexts using a quantitative framework

**DOI:** 10.3389/fgeed.2026.1759382

**Published:** 2026-08-14

**Authors:** Christopher R. Handelmann, Erin Skeens, George P. Lisi, Michael J. Buck

**Affiliations:** 1 Department of Biochemistry, Jacobs School of Medicine and Biomedical Sciences, State University of New York at Buffalo, Buffalo, NY, United States; 2 Department of Biomedical Informatics, Jacobs School of Medicine and Biomedical Sciences, State University of New York at Buffalo, Buffalo, NY, United States; 3 Department of Molecular Biology, Cell Biology and Biochemistry, Brown University, Providence, RI, United States; 4 Brown RNA Center, Brown University, Providence, United States

**Keywords:** Cas12a, Cas9, chromatin, CRISPR, nucleosome

## Abstract

Chromatin presents a significant obstacle to CRISPR-Cas gene editing, as chromatin restricts nuclease access to DNA. Recent advances have produced a wide range of high-fidelity Cas9 and Cas12a variants with enhanced properties. However, their precision in targeting DNA within different contexts remains poorly understood. This gap limits our ability to predict and optimize Cas performance in the dynamic chromatin landscape. To elucidate how chromatin variability impacts Cas editing accuracy, we utilized GEMiNI-seq to systematically profile wild-type and engineered Cas9 and Cas12a nucleases across a range of nucleosome sequences. All nucleases showed reduced cleavage in nucleosomal DNA relative to naked DNA, with the strongest inhibition at dyad-proximal sites. Cleavage within nucleosomes was highly variable, with wtSpCas9 exhibiting up to 65-fold different activity depending on the nucleosome type. Editors with high catalytic activity (wtSpCas9, HIFIv2, LbCas12a ULTRA) consistently outperformed high-fidelity variants such as evoSpCas9, which displayed excellent specificity on naked DNA but poor performance in nucleosomal contexts. ROC and PRC analyses revealed that nucleosome sequence and orientation shape both sensitivity and specificity, with HIFIv1 emerging as the top-performing nuclease for nucleosomal targets, while evoSpCas9 excelled in exposed contexts. Our findings demonstrate that local nucleosome sequence and structure profoundly influence Cas nuclease accessibility and specificity. Variability in cleavage across nucleosome types underscores the need to consider chromatin context during target selection and nuclease design. These results provide a framework for selecting or engineering Cas editors optimized for therapeutic genome editing within chromatin.

## Introduction

The ability of CRISPR-Cas systems to introduce site-specific DNA double-strand breaks has revolutionized genome editing (Jinek et al., 2012; Cong et al., 2013), but targeting DNA within chromatin presents a significant barrier to efficient editing (Horlbeck et al., 2016; Yarrington et al., 2018). Nucleosomes, the fundamental unit of chromatin, limit access to targets by tightly wrapping ∼147 base pairs (bp) of DNA around a histone octamer, creating a barrier to Cas nucleases. Among the most widely used CRISPR-Cas systems, *Streptococcus pyogenes* Cas9 (SpCas9) and Cas12a (also known as Cpf1) differ in their structural organization, Protospacer Adjacent Motif (PAM) requirements, and mechanisms of cleavage, providing unique opportunities and limitations when targeting nucleosomal DNA (Jinek et al., 2012; Zetsch et al., 2015; Marraffini, 2015; Kim et al., 2016; Mojica et al., 2009; Babu et al., 2021; Huai et al., 2017; Cofsky et al., 2020). Assessing how Cas nucleases engage nucleosome-bound targets is critical for developing improved Cas editors and for reducing the potential of off-target editing (Zhang et al., 2015).

Previous studies have shown how nucleosomes impact Cas’ ability to access and cleave target sequences. Researchers leveraged the artificial Widom-601 nucleosome and targeted SpCas9 to various existing “NGG” PAM sequences throughout the nucleosome (Horlbeck et al., 2016; Hinz et al., 2015; Isaac et al., 2016; Nagamura et al., 2024). SpCas9 cleavage depends on PAM accessibility across the nucleosome. Targets located near the dyad are strongly inhibited, while PAMs positioned near the nucleosome edge show partial inhibition (Hinz et al., 2015; Isaac et al., 2016; Handelmann et al., 2023). In contrast, exposed PAMs support efficient cleavage even when the target sequence is occluded within the nucleosome. The ability of the AsCas12a nuclease to digest nucleosomal targets showed similar inhibition as seen for SpCas9 (Strohkendl et al., 2021). AsCas12a also showed a reduced ability to digest targets positioned outside of the nucleosome, in the linker region, showing that wtAsCas12a had reduced ability to contend with the nucleosome structures compared to SpCas9 (Strohkendl et al., 2021). Different nucleosome positioning sequences (NPSs) have also been shown to influence Cas nuclease cleavage. The ability of SpCas9 to cleave targets in the Widom-601 NPS, compared to the weaker, more biologically relevant 5S NPS, resulted in more target digestion at the edge of the 5S nucleosome but similar inhibition of SpCas9 cleavage at the dyad (Isaac et al., 2016). Structural studies on SpCas9 binding to the nucleosome further showed the importance of the PAM-Interacting Domain (PID) for accessing the nucleosome target, and also the hindrance that the histone octamer core poses to SpCas9 being able to undergo its complete conformational changes when cleaving a target (Nagamura et al., 2024). The inhibition of nucleases within nucleosomes reduces nucleosomal target cleavage *in vivo*, with increased off-targeting to other sequences demonstrated *in vitro* (Handelmann et al., 2023; Uusi-Mäkelä et al., 2018; Kallimasioti-Pazi et al., 2018).

Improving the specificity of CRISPR-Cas editing has become a significant focus in the field, with numerous variants being designed to enhance specificity while maintaining on-target activity for SpCas9 (Casini et al., 2018; Vakulskas et al., 2018; Slaymaker et al., 2016; Nathaniel Hunter Roberts and Anthony Vakulskas, 2023) and Cas12a (Zetsche et al., 2015; Kim et al., 2016; Zhang et al., 2021; Huang et al., 2022). These modified nucleases incorporate amino acid substitutions that dampen non-specific interactions, restrict conformational transitions required for cleavage, or increase nuclease on-target activity, thereby reducing off-target activity while maintaining normal target digestion (Huai et al., 2017; Casini et al., 2018; Vakulskas et al., 2018; Slaymaker et al., 2016; Zhang et al., 2021; Huang et al., 2022). The utility of high-fidelity nucleases is especially relevant for editing within chromatin, where the constraints imposed by nucleosomes can obscure targets and exacerbate off-target cleavage. Assessing the performance of high-fidelity nucleases in nucleosomal contexts is therefore essential for evaluating their practical utility across various applications and for elucidating the interplay between Cas nuclease modifications and their impact on cleaving occluded targets.

To define the optimal Cas9 or Cas12 editor for targeting nucleosomal sites, we used the Gene Editor Mismatch Nucleosome Inhibition (GEMiNI) assay. Using GEMiNI-seq, we compared wild-type SpCas9, AsCas12a, and LbCas12a, along with engineered variants—evoSpCas9, HIFIv1, HIFIv2, AsCas12a ULTRA, and LbCas12a ULTRA on their ability to accurately target sites located anywhere within three nucleosome positioning sequences (NPSs), Widom-601, 5S, or MMTV. By including off-target sequences, GEMiNI-seq allows simultaneous evaluation of Cas fidelity at linker and nucleosome-targeted sites. The results provide a comprehensive view of editing efficiency and fidelity across diverse nucleosome contexts.

## Results

### Nucleosome-DNA interactions drive Cas RNP accessibility

**FIGURE 1:**
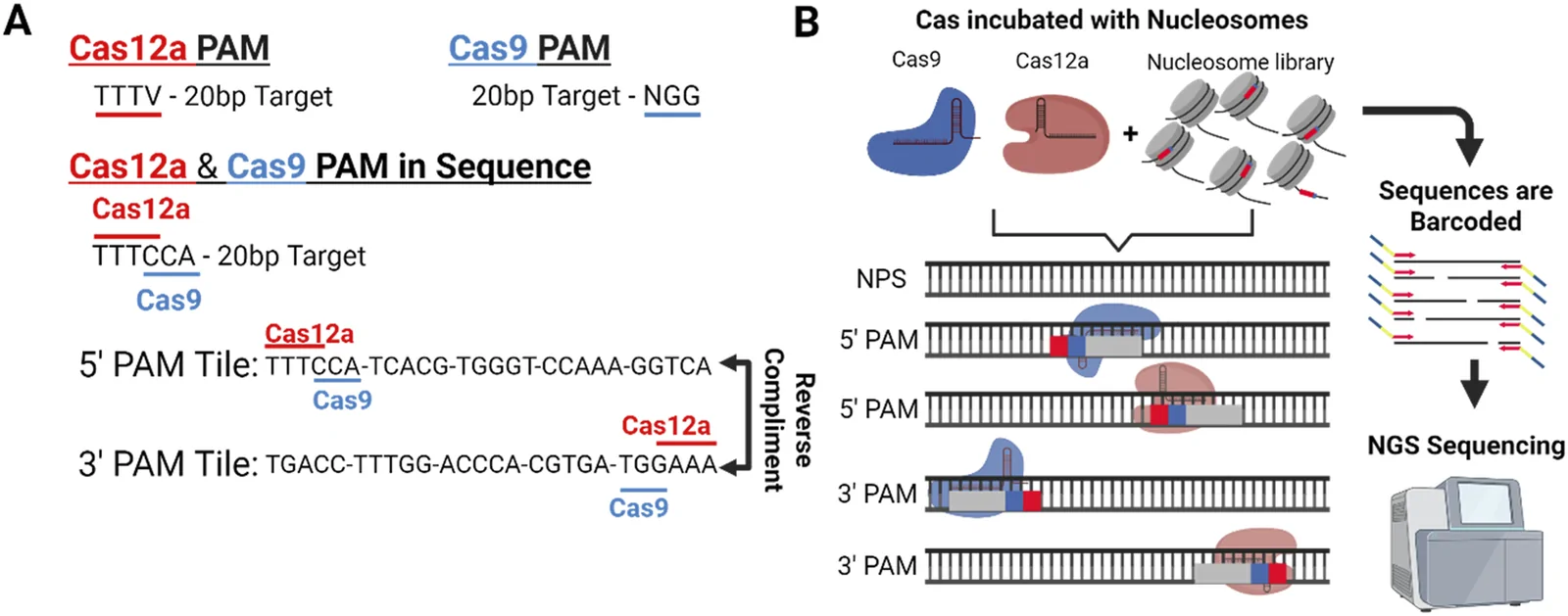
Gemini-seq library design and experimental procedure. **(A)** The Cas target sequence is designed with the canonical SpCas9 PAM (NGG) and the canonical Cas12a PAM (TTTV), shown in blue and red respectively, aligned in sequence adjacent to a 20-nt target. The sequential PAM is positioned at both the 5′ and 3′ ends of a 20-bp DNA sequence. **(B)** A 20-bp DNA sequence and a 6-bp sequential PAM sequence (26 bp total length) are tiled at every base pair position through a nucleosome positioning sequence (NPS) and into the linker. The sequential PAM and target sequence are tiled in both the 5′ and 3′ orientations, generating a library of NPS sequences. The DNA library was formed into nucleosomes and purified, forming a nucleosome library. Both the nucleosome and the DNA library were separately incubated with Cas9 (blue) and Cas12a (red) variants targeting the 20-bp target sequence. Samples are then indexed with two-step PCR and sequenced.

To compare the ability of Cas9 and Cas12a to target chromatin, we tailored the GEMiNI-seq assay with a dual-target sequence containing both the Cas9 “NGG” and the Cas12a “TTTV” PAMs in sequence adjacent to a 20-nt target sequence, allowing Cas editors that bind either PAM to target the same sequence (Figure 1A). These target sequences were positioned at every base position throughout the well-defined NPSs of Widom-601, 5S, and MMTV-NucA (Yu and Buck, 2020; Chen et al., 2017; Lowary and Widom, 1998; Richard-Foy and Hager, 1987; Hayes and Wolffe, 1993; Dong et al., 1990; Chen et al., 2014), with the PAM oriented both 5′ and 3′ of the 20-nt sequence, giving both conformations of the target and PAM relative to the nucleosome (Figure 1B). The library of NPS DNA sequences is formed into nucleosomes and incubated with Cas9 and Cas12a nuclease variants. The number of each uncleaved sequence remaining in the sample pool, quantified by Next-Generation Sequencing, enables a robust analysis of the different capacities of various Cas editors to edit within nucleosomal structures (Figure 1B).

**FIGURE 2:**
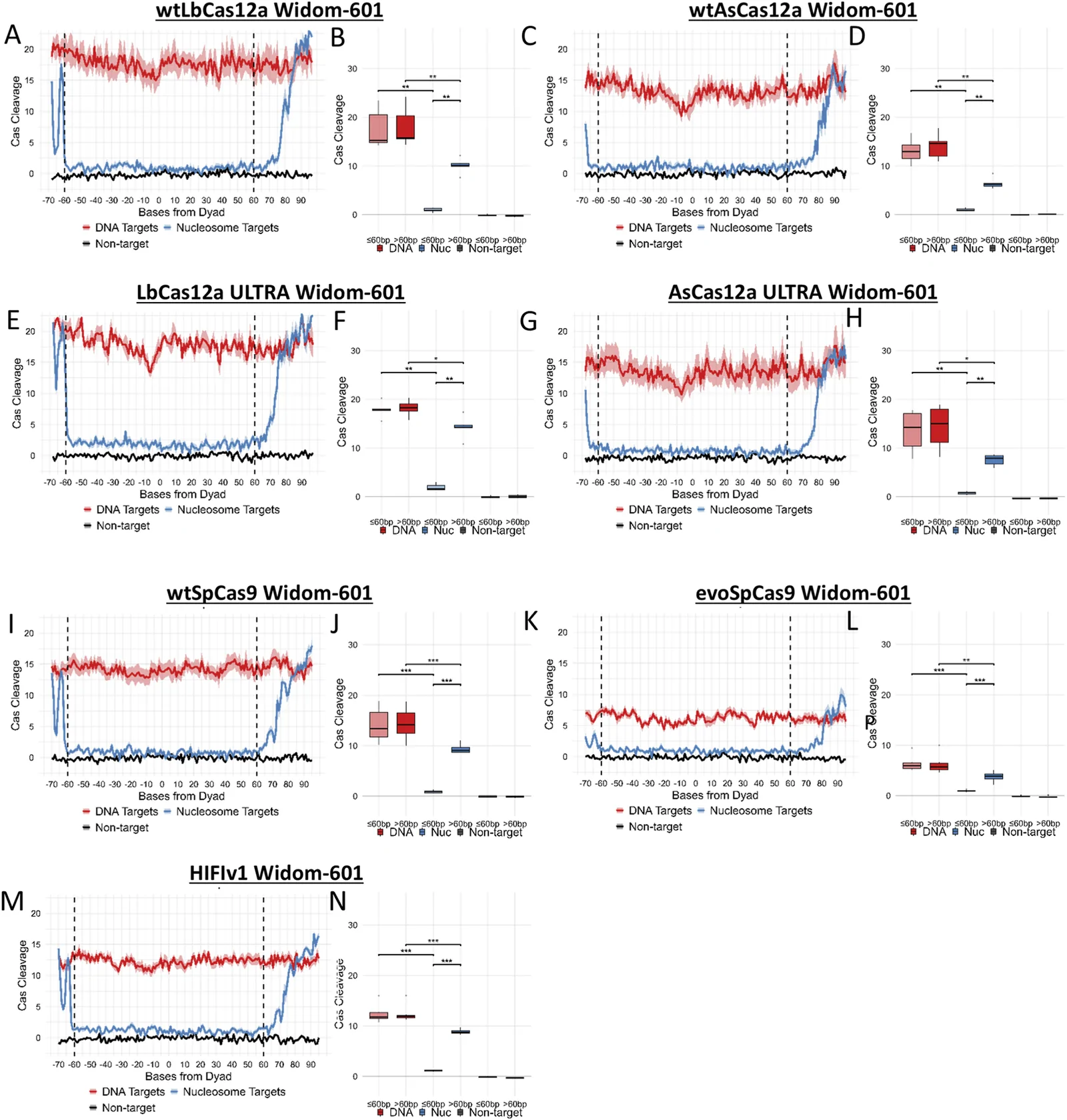
The Widom-601 NPS shows a landscape of varying accessibility for different Cas editors. The digestion of the Widom-601 NPS 3′ PAM at every base pair by **(A)** wtLbCas12a, **(C)** wtAsCas12a, **(E)** LbCas12a ULTRA, **(G)** AsCas12a ULTRA, **(I)** wtSpCas9, **(K)** evoSpCas9, and **(M)** SpCas9 High Fidelity variant 1 (HIFIv1) within a nucleosome, on naked DNA, and on a non-target sequence. Black dashed lines show the SHL position for −6 and 6, 60 base pairs from the dyad. The ribbons within the line graphs are the standard error of the mean for the 5 biological replicates of each Cas editor. **(B,D,F,H,J,L,N)** Each Cas editor’s digestion is plotted for positions within the nucleosome (SHL -6 to SHL 6) and in the linker (positions beyond SHL -6 and SHL 6) for the naked DNA, nucleosome, and non-target sequences. P-values were calculated using two-sided Wilcoxon rank-sum (Mann–Whitney) adjusted using the Benjamini–Hochberg procedure. *P < 0.05, **P < 0.01, ***P < 0.001, ****P < 0.0001. All graphs represent 5 biological replicates.

To assess the affinity of Cas editors for target sequences within the nucleosome landscape, we tested multiple Cas editors with five distinct target sequences (Figure 2). Exposed targets exhibit a greater capacity for cleavage relative to targets within Widom-601 (Figure 2). The Widom-601 NPS forms a strong barrier to Cas editing within 60 bp of the dyad, though a difference in Cas editing is shown, with the highly active Cas editors such as LbCas12a ULTRA having a greater amount of target cleavage at the nucleosome edge and into the nucleosome (Figures 2E,F). To further elucidate the ability of Cas editors to access the nucleosome, the Cas editors were targeted at a single sequence at varying concentrations (Supplementary Figure S1). The amount of target cleavage increases with Cas editor concentration, both for targets within the linker and targets within the 601 nucleosome, but the nucleosome structure significantly impaired the effect of RNP concentration on target digestion (Supplementary Figure S1). The various Cas editors have greater cleavage of the targets within the 5S and MMTV nucleosomes than in Widom-601, with wtSpCas9, LbCas12a ULTRA, and AsCas12a ULTRA still showing the most cleavage (Supplementary Figures S2–S5). The 5’ (left side) of the 5S nucleosome showed the most digestion by the Cas editors, both in the 5′ PAM targets and in the 3′ PAM target orientation (Supplementary Figures S2–S5). The MMTV nucleosome showed better editing by both orientations on the 3’ (right side) of the nucleosome. The 5S and MMTV NPS, while still greatly reducing Cas cleavage, are a more permissive barrier, with greater cleavage occurring within 60 bp of the dyad.

### Cas editor K_C_ throughout nucleosome positioning sequence

**FIGURE 3:**
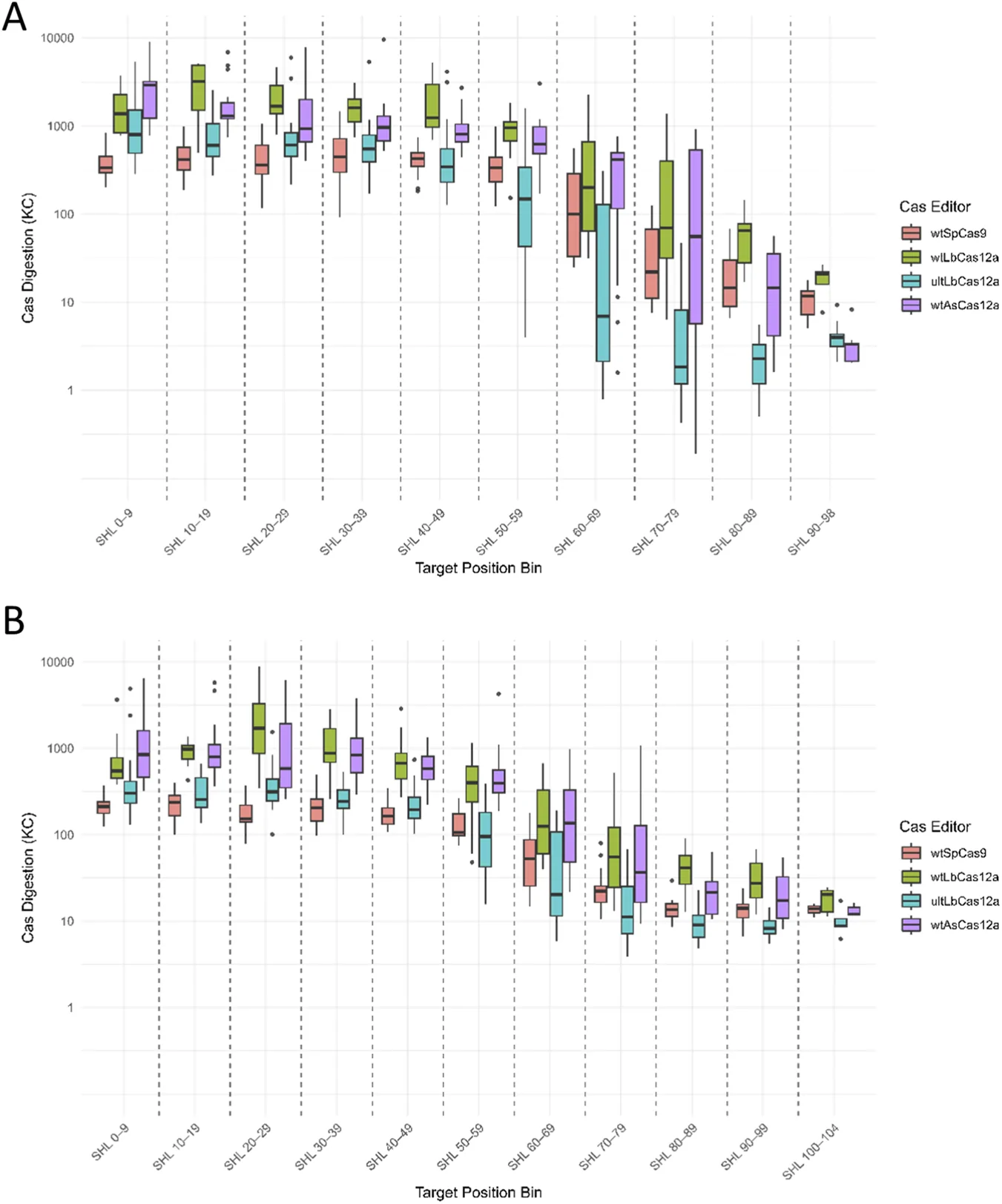
The amount of Cas RNP needed to achieve target cleavage varies across the Widom-601 and 5S nucleosomes and differs between Cas editors. Kc values (enzyme concentration required to reach 80% cleavage, derived from nonlinear regression fits) are plotted for each Cas editor at 10 bp intervals from the dyad out into the linker for **(A)** the Widom-601 and **(B)** the 5S NPS. Each section represents the Kc values for both 5′ and 3′ PAM-oriented targets, with Kc values grouped by SHL and plotted on a logarithmic scale to capture the wide dynamic range across nucleosomal positions. Higher Kc values indicate positions that require more Cas RNP to achieve target cleavage, indicative of decreased digestion, whereas lower values denote more accessible targets. All Cas editors are composed of 3 biological replicates.

To characterize the cleavage kinetics of Cas RNPs, dependent on both the binding and catalytic steps within nucleosomes, we estimate the K_C_ value by fitting cleavage data across varying RNP concentrations using nonlinear regression, analogous to the calculations for dissociation constants (K_D_) (Yu et al., 2019). To compare the amount of each editor required to cleave a given nucleosomal target, the K_C_ for each Cas editor is grouped into 10-base-pair bins, dividing the NPS. The K_C_ values clearly showed the trend that targets located closer to the dyad would require far more Cas RNP to achieve the same amount of cleavage as a target located farther from the dyad, with decreasing K_C_ values for targets located beyond 60 bp (Figure 3; Supplementary Figures S6, S7; Supplementary Table S1). The editors that required less RNP to cleave exposed targets also required less to cleave targets in all nucleosome contexts, indicating a consistent hierarchy based on the editor cleavage activity (Figure 3A). The hierarchical order of the Cas editors also persisted across Widom-601, 5S, and MMTV (Figures 3A,B; Supplementary Figures S6, S7). LbCas12a ULTRA, AsCas12a ULTRA, wtSpCas9, HIFIv1, and HIFIv2 have consistently lower K_C_ values. LbCas12a ULTRA has the lowest K_C_ needed to cleave targets from 70 to 40 bp away from the dyad, indicating a potentially favorable ability to compete with the histone octamer. In contrast, evoSpCas9 consistently has the highest K_C_ across all nucleosome regions. Both Cas12a ULTRA variants outperform their wild-type progenitors in the RNP required to edit either exposed or nucleosome-bound targets. K_C_ values binned across the NPS show a strong positional effect, with targets near the dyad needing more Cas RNP for cleavage and K_C_ dropping beyond ∼60 bp, with the more active Cas editors requiring less RNP across all contexts. The two defining factors for nucleosomal cleavage efficiency are the target’s proximity to the dyad and editor activity.

### Cas nuclease mismatch tolerance

To more comprehensively assess how each nuclease tolerates off-targeting, a nucleosome library was designed with 29 potential target sequences with varying levels of complementarity (Supplementary Table S2). Guide RNAs were generated to match five of the sequences, providing a variety of sequence compositions to assess target binding and cleavage. The other twenty-four targets have a varying number of mismatches, resulting in a range from single-base mismatches to no complementarity. This design enables a comprehensive analysis of nuclease tolerance across different targets and chromatin states. The targets were tiled through various NPS sequences (Supplementary Table S3).

**FIGURE 4:**
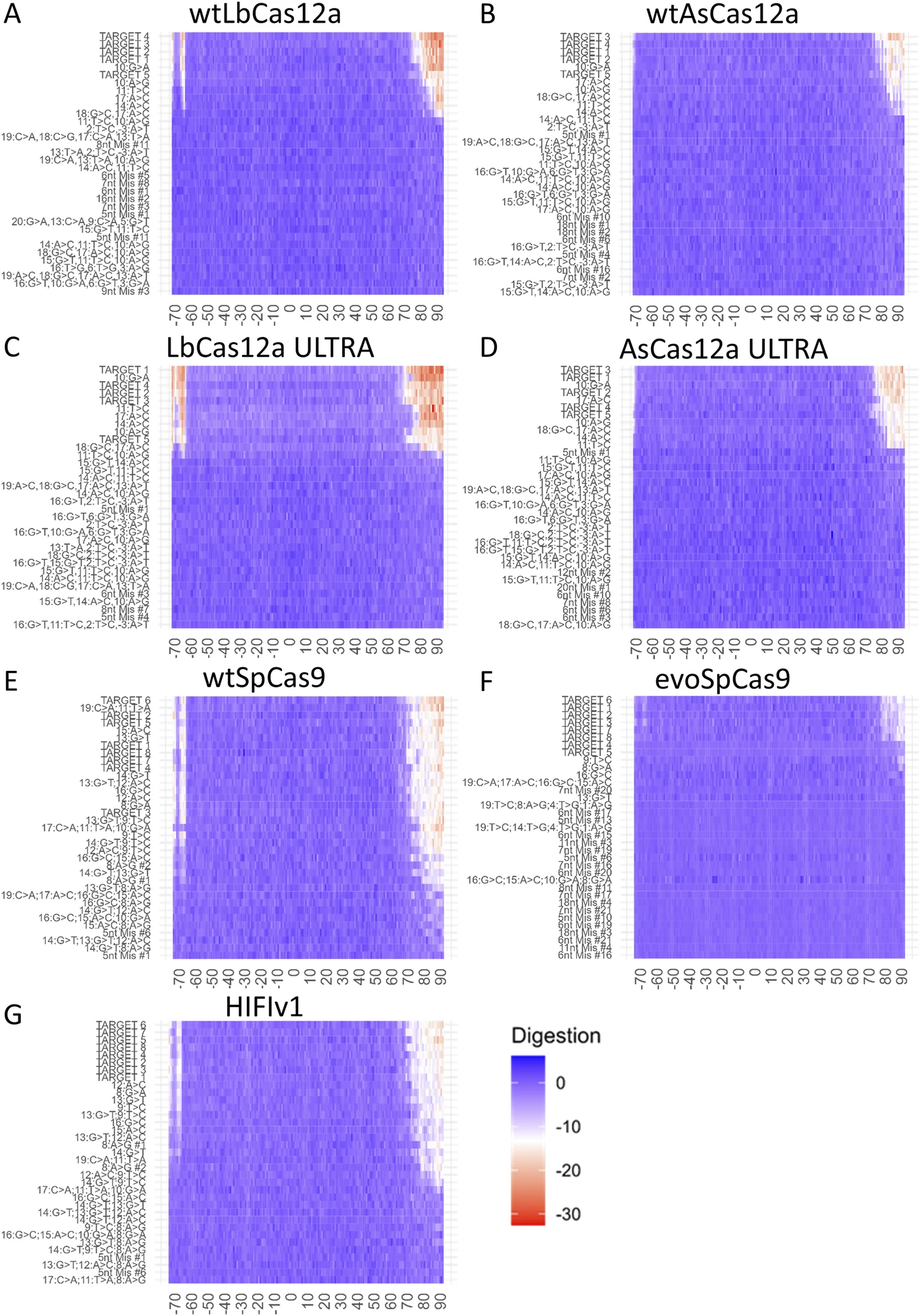
Cas nuclease tolerance for cleaving various sequences within the Widom-601 nucleosome. The digestion of the Widom-601 nucleosome positioning sequence at every base pair by **(A)** wtLbCas12a, **(B)** wtAsCas12a, **(C)** LbCas12a ULTRA, **(D)** AsCas12a ULTRA, **(E)** wtSpCas9, **(F)** evoSpCas9, **(G)** and HIFIv1. The order of the targets on the y-axis is determined by averaging the amount of cleavage of +50 bp to +95 bp from the dyad. The top 35 ranked sequences are shown, with full heat maps containing all sequences provided in the supplemental figures. All digestions were performed with 30 nM of each Cas nuclease on a 3 nM nucleosome library concentration. Amount of digestion is shown as a heatmap transitioning from blue (not digested) to red (digested).

With multiple on and off-target sequences, some nucleases had greater cleavage of off-target sequences relative to the on-target sequences, but only in exposed DNA. When the potential target was within the nucleosome, the tolerance for targets with mismatching bases dropped, while on-target cleavage of targets on the edge was less impaired (Figures 4A–G; Supplementary Figures S8–S14). The LbCas12a ULTRA modification exhibits the greatest cleavage on exposed targets and digests further into the nucleosome for both on-targets and some mismatches (Figure 4C; Supplementary Figure S10). The LbCas12a ULTRA modifications did not induce greater cleavage on new off-target sequences but increased the amount of cleavage on the off-targets already tolerated by the wtLbCas12a nuclease (Figures 4A,C; Supplementary Figure S8; Supplementary Figure S10). AsCas12a ULTRA also increases the amount of digestion seen from the wtAsCas12a nuclease, though both AsCas12a nucleases show reduced cleavage compared to the LbCas12a nucleases (Figures 4A–D; Supplementary Figures S8–S11).

The best nucleases for target specificity, as demonstrated by the grouping of target sequences at the top of the heat map, are evoSpCas9 and HIFIv1 (Figures 4F,G; Supplementary Figures S13, S14). Both variants have improved targeting compared to wtSpCas9, which has a high tolerance for cleaving off-targets, tolerating up to 3 base-pair mismatches (Figure 4E; Supplementary Figure S12). The variant with the highest fidelity is evoSpCas9, which groups all on-target cleavages together (Figure 4F; Supplementary Figure S13). There is some tolerance for single-base mismatches at the 8th and 9th bases from the PAM that evoSpCas9 cleaves (Figure 4F; Supplementary Figure S13). The high fidelity of evoSpCas9 results in significantly reduced activity (Spasskaya et al., 2023), with low levels of target cleavage observed in both nucleosomal linkers and naked DNA (Figure 4F; Supplementary Figure S13). The evoSpCas9 editor shows the lowest cleavage within nucleosomes, highlighting the need for activity to access these targets. By comparison, the HIFIv1 SpCas9 performs better in nucleosomes (Figure 4G; Supplementary Figure S14). HIFIv1 maintains greater cleavage of nucleosome-bound targets, while the reduced tolerance for mismatches shows improved fidelity over wtSpCas9, though off-target cleavage still occurs. (Figure 4G; Supplementary Figure S14). The digestion results show that off-target cleavage occurred primarily on exposed DNA, while mismatch tolerance declined sharply for nucleosome-embedded targets. These results demonstrate a trade-off between a Cas enzyme’s intrinsic activity, which increases its ability to access nucleosome-bound targets, and increased mismatch tolerance.

### Cas editor specificity and sensitivity

**FIGURE 5:**
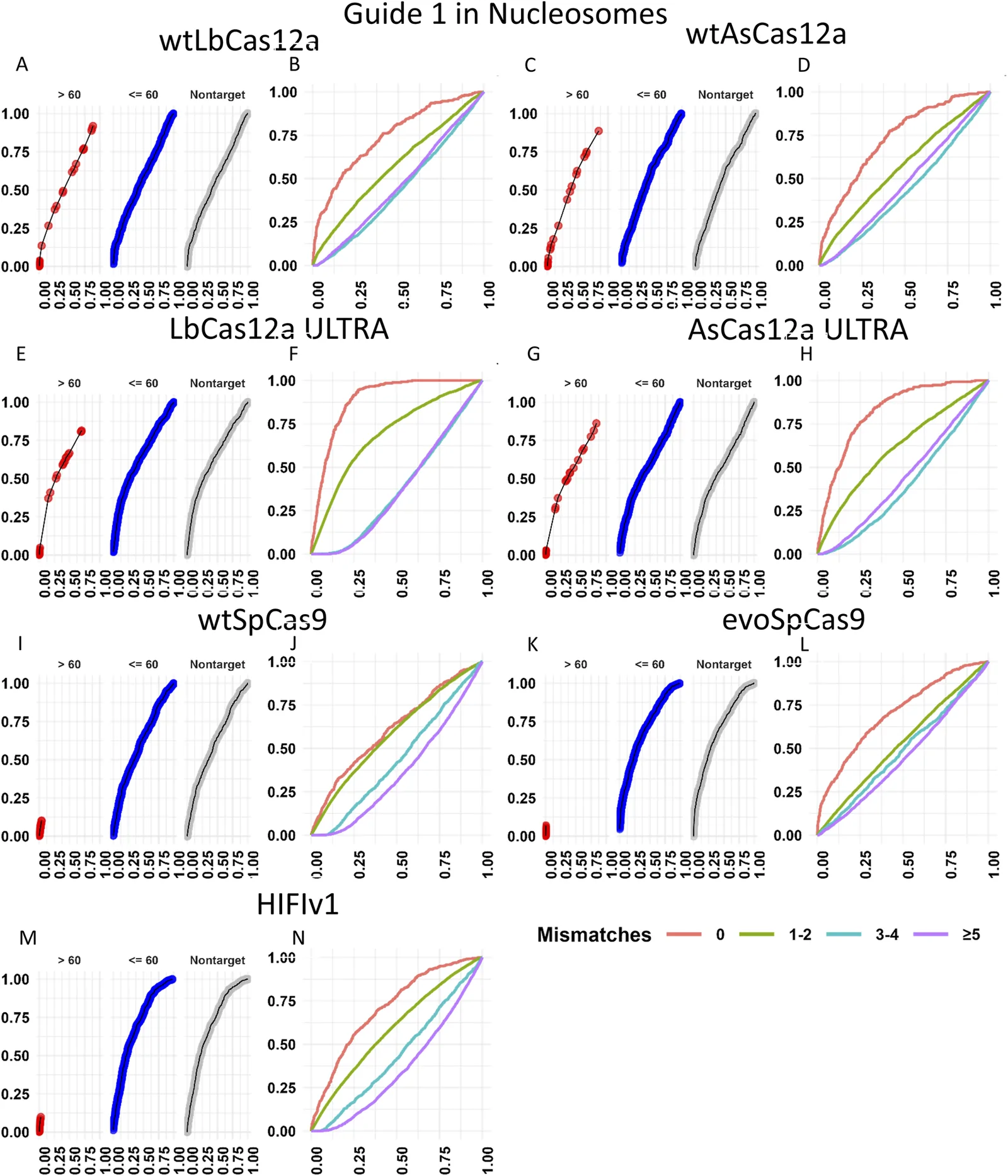
Assessing the specificity and sensitivity of each Cas editor. The 7,500-sequence library was used to evaluate the performance of each Cas editor. Sequences were ranked by cleavage efficiency, and true positive rate (TPR) versus false positive rate (FPR) was calculated to generate receiver operating characteristic (ROC) curves for **(A)** wtLbCas12a, **(C)** wtAsCas12a, **(E)** LbCas12a ULTRA, **(G)** AsCas12a ULTRA, **(I)** wtSpCas9, **(K)** evoSpCas9, and **(M)** HIFIv1. Each ROC curve is subdivided into targets within ±60 bp of the dyad (blue), targets outside ±60 bp (red), and mistarget sequences (green). The cumulative frequency for the target sequences (pink) and varying amounts of mismatches (1–2 (green), 3–4 (blue), and 5+ (purple)) for the Cas12a variants **(B,D,F,H)** and the Cas9 variants **(J,L,N)**. A greater separation between target and mismatch curves indicates higher specificity. All Cas RNP digestions were at 30nM–3 nM nucleosome library.

To rigorously assess and compare the specificity and sensitivity of distinct Cas editors, target cleavage efficiencies were quantitatively ranked against a panel of potential off-target sequences. This enabled the calculation of specificity and sensitivity, which were subsequently used to generate Receiver Operating Characteristic (ROC) curves for each guide editor pair (Figure 5; Supplementary Figures S15–S23). These plots provide an intuitive visualization of editor specificity and reveal how editing efficiency is impacted between naked DNA and nucleosomal DNA. The ROC plots are further stratified by target class, including linker targets, nucleosomal targets, and targets containing mismatches.

Among the editors tested, HIFIv1, LbCas12a ULTRA, and wtSpCas9 showed superior sensitivity and specificity within nucleosomal regions, as evidenced by their favorable ROC curves for nucleosomal targets (Figures 5E,I,M). These editors exhibited high cleavage rates for targets located more than 60 bp from the nucleosomal dyad, clustering in the lower-left quadrant of the ROC space—indicative of strong true positive discrimination with minimal off-target activity. EvoSpCas9 exhibited the greatest specificity on naked DNA, where its ROC curve reflected high sensitivity and precise discrimination between on-target and mismatch-containing sequences (Supplementary Figures S19–S23).

Mismatch tolerance can be further evaluated through cumulative frequency distributions that track the prevalence of target mismatches across the FPR axis. When a mismatch frequency is close to the target frequency, it reflects a reduced specificity for that mismatch. The cumulative frequencies for mismatches in LbCas12a ULTRA and HIFIv1 show greater separation from the target frequency and thus better specificity (Figures 5F,N; Supplementary Figure S15–S23). The wtLbCas12a, wtAsCas12a, and AsCas12a ULTRA show poorer specificity, with mismatch frequency, particularly for 1-2 base mismatches, being closer to the target frequency (Figures 5B,D,H; Supplementary Figures S15–S23). The evoSpCas9 nuclease shows the least mismatch tolerance, with a markedly lower tolerance for sequences with 1-2 base mismatches compared to the other editors (Figure 5L; Supplementary Figures S15–S23).

The quantification for each Cas editor’s specificity can be reduced to the area under the ROC (AUROC), offering a direct comparison of editor specificity (Supplementary Table S4). The AUROC scores show that target accessibility has a dramatic impact on performance. The greatest impact was observed for evoSpCas9, which exhibited excellent specificity for naked DNA but poorer performance on nucleosomal DNA due to restricted access, even in linker regions (Figure 4F). Overall, SpCas9 variants outperform Cas12a variants in naked DNA, whereas in nucleosomal DNA, specificity is more tightly coupled to Cas editor cleavage activity. Precision-Recall Curves (PRCs) provide an additional metric for evaluating specificity by controlling for the imbalance between targets and nontargets in the nucleosome library. Quantifying the area under the PRC (AUPRC) yields results very similar to those of AUROC for Cas editor specificity, with editors with high AUROC also having higher AUPRC. The ROC and PRC analyses show that target accessibility is a dominant determinant of specificity and sensitivity, with chromatin favoring activity over fidelity in editing, with highly active editors outperforming ultra-high-fidelity variants.

### Ranking nucleases

**FIGURE 6:**
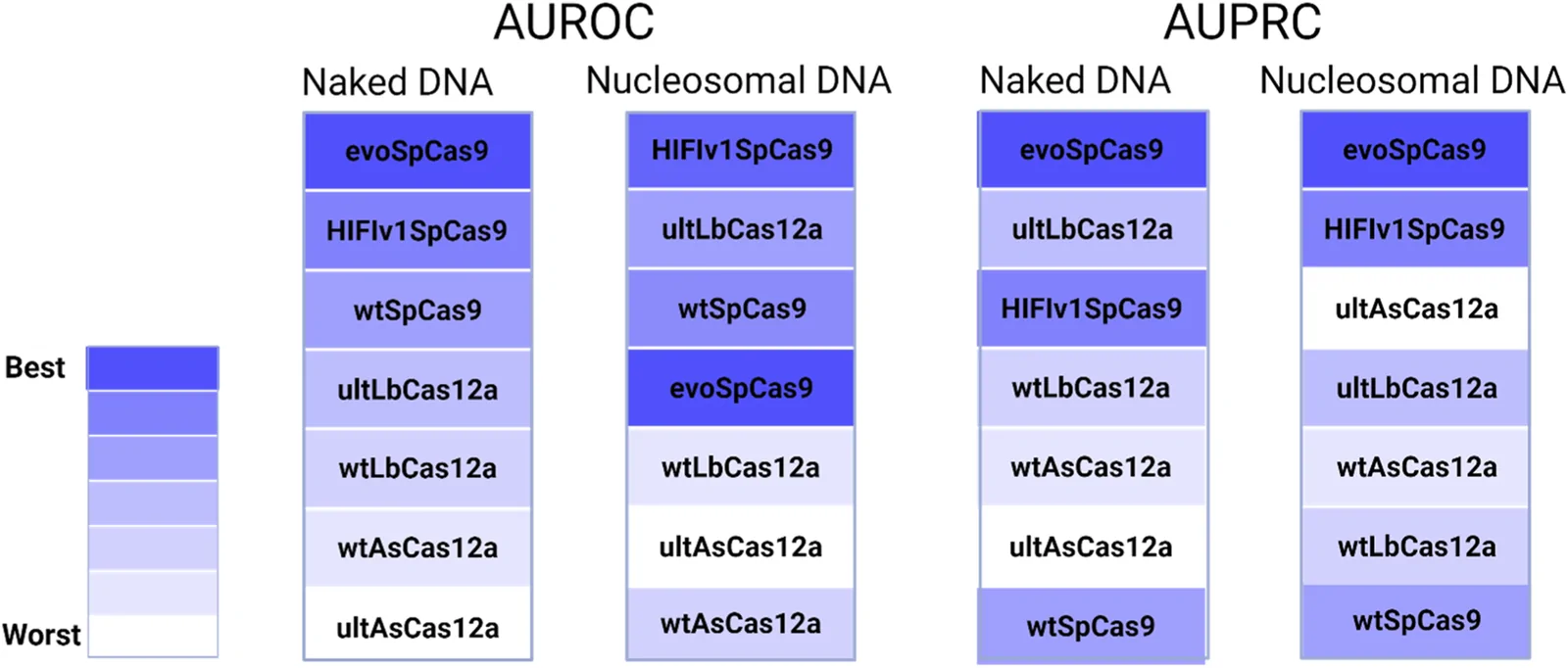
Ranking the editors based on AUROC and AUPRC. Each editor tested is ranked based on the area under the ROC (AUROC) and area under the Precision-Recall curve (AUPRC). The AUROC and AUPRC scores are calculated for each Cas nuclease cleavage within both naked DNA and nucleosomes. The editors are listed from top to bottom by AUROC or AUPRC values in Supplementary Table S4, with higher values indicating better performance and a higher ranking. The color for each Cas editor is depicted on a scale from blue to white based on Cas editor performance, as measured by AUROC in naked DNA, to show the change in order based on nucleosome context and the similarity in ranking between AUROC and AUPRC. The AUROC or AUPRC ranking of each Cas editor is the average of 5 biological replicates.

To thoroughly evaluate and compare the specificity and sensitivity of different Cas editors, target cleavage efficiencies were quantitatively ranked using AUROC and AUPRC scores for multiple on- and mismatching targets (Supplementary Table S4). The difference in Cas nucleases’ rankings reflects their ability to cleave the intended targets (captured by AUROC) and their tolerance for off-target activity (captured by AUPRC). The AUROC and AUPRC scores provide a clear measure of editor specificity, showing how editing efficiency differs between naked DNA and nucleosomal DNA. Thus, we can rank all tested editors based on their AUROC and AUPRC scores. The best editor in naked DNA is evoSpCas9, with the highest scores for both AUROC and AUPRC (Figure 6). The best editor in nucleosomes is HIFIv1 based on AUROC or evoSpCas9 by AUPRC (with HIFIv1 ranking second for AUPRC) (Figure 6).

## Discussion

This study is the first to directly compare CRISPR-Cas nucleases in their ability to accurately target DNA sites embedded within nucleosomes. We developed a novel approach using a composite PAM-target sequence compatible with both Cas9 and Cas12a, enabling direct comparison of these nucleases on overlapping target sites. To assess variability in nucleosome-mediated inhibition and editing specificity, we designed a nucleosome library containing multiple positioning sequences with embedded off-target sites. This allowed us to systematically evaluate both the sensitivity and specificity of target selection across various nucleosomal contexts.

Across all CRISPR-Cas nucleases tested in our study, we show that nuclease cleavage decreases as the target sequence approaches the dyad. This pattern is consistent across all tested nucleosome positioning sequences, although the extent of inhibition varies between nucleosome types. We used three nucleosome positioning sequences in this study: the synthetic Widom-601 with two naturally occurring sequences from 5S and MMTV (Yu and Buck, 2020; Chen et al., 2017; Lowary and Widom, 1998; Richard-Foy and Hager, 1987; Hayes and Wolffe, 1993; Dong et al., 1990; Chen et al., 2014). The Widom-601 sequence is extremely well-studied and has been used in over 100 structural studies. Widom-601 forms nucleosomes *in vitro* with high efficiency in a single predominant position, yielding reproducible, well-defined structures (Yu and Buck, 2020). 5S and MMTV nucleosomes are biologically derived sequences that have been used for various studies (Thiriet and Hayes, 1998; Frouws et al., 2016). 5S and MMTV-nucA nucleosomes exhibited higher cleavage for the Cas nucleases tested within 60 bp of the nucleosomal dyad than the Widom-601. For editing the edge within the nucleosome (≥60 BP of the dyad), wtSpCas9 demonstrated greater cleavage in 5S compared to Widom-601, with up to 15 times greater cleavage, and up to 65 times greater in MMTV (Supplementary Table S5). The disparity in cleavage among different nucleosome targets increased with higher activity nucleases, with LbCas12a ULTRA showing up to 282-fold greater cleavage in 5S and up to 224-fold in MMTV when compared to Widom-601. Given that 75%–90% of the eukaryotic genome is wrapped into nucleosomes, heterogeneity in cleavage between NPSs would result in significant differences in Cas editing efficiency and specificity, depending on the target context.

A complex relationship between PAM orientation and the distance from the dyad has been shown to influence access to the nucleosome (Hinz et al., 2015; Isaac et al., 2016; Handelmann et al., 2023; Makasheva et al., 2021). This pattern is present throughout our dataset for all of the NPS. The Widom-601 NPS exhibits this inhibition in the linker, where the rotation of the PAM facing toward the histone octamer reduces nuclease cleavage. The 5S and MMTV NPS exhibit a pattern of cleavage from PAM rotation within 60 bp of the dyad nucleosome, which is indistinct within 60 bp of the Widom-601 dyad. The cleavage pattern was more distinct on the 5’ (left side) of the 5S nucleosome and on the 3’ (right side) of the MMTV-nucA nucleosome. The amount of rotational inhibition varies among the different Cas nucleases we tested. The evoSpCas9 nuclease, with the lowest activity, shows similar inhibition for binding to linker and nucleosome targets regardless of PAM orientation. The Cas12a nucleases wtAsCas12a and LbCas12a ULTRA exhibit the highest cleavage of naked DNA and are more affected by PAM orientation. The wtAsCas12a shows very clear inhibition from the PAM orientation in the Wiodm-601 linker, highlighting the importance of PAM exposure for its activity. Conversely, LbCas12a ULTRA shows the least inhibition by PAM rotation in Widom-601, with high cleavage across all targets beyond the nucleosome edge. When targeting within the 5S nucleosome, both wtAsCas12a and LbCas12a ULTRA display a distinct cleavage pattern based on PAM orientation, with increased cleavage when the PAM faces away from the histone octamer. Our results show that the interaction of PAM rotation on nuclease cleavage is a complex relationship, depending on both the local affinity of the NPS for the histone octamer core and the affinity of the Cas nuclease for its target.

LbCas12a ULTRA and AsCas12a ULTRA have improved digestion on the nucleosome edge compared to their wtCas12a origins. This can be driven by the mutations in the Cas12a PID, which have been previously shown to increase PAM binding strength and subsequently increase target cleavage (Zhang et al., 2021; Huang et al., 2022), making the nuclease better able to compete with the nucleosome once bound to the PAM, driving higher target cleavage. Our GEMiNI-seq results show that evoSpCas9 exhibits low levels of target digestion in both naked DNA and nucleosomes, consistent with previous reports (Casini et al., 2018; Spasskaya et al., 2023). This reduced activity aligns with prior findings that high-fidelity Cas editors often have decreased editing efficiency due to inherent trade-offs between specificity and catalytic function (Schmid-Burgk et al., 2020). We demonstrate that evoSpCas9 shows high specificity for naked DNA targets but displays poor specificity within nucleosomes, driven by its inability to contend with the nucleosome and cleave target sequences within the nucleosome or the adjacent linker. The best editor for nucleosomal targets is the HIFIv1 SpCas9 variant, which can compete with the nucleosome for target cleavage while maintaining high affinity for the target sequence. The wtSpCas9 showed high accessibility across the various nucleosomes but was more tolerant of mismatches, thereby reducing its fidelity.

Determinants of differential Cas activity at nucleosomes, especially between Cas9 and Cas12a, may be nuclease size and the mechanism of target engagement. SpCas9 is modestly larger than AsCas12a and LbCas12a, which likely increases steric hindrance for deeply embedded targets, whereas the smaller Cas12a enzymes may experience slightly reduced geometric constraints at edge or outward-facing PAM positions (Cheng et al., 2021; Paul and Montoya, 2020). SpCas9 and Cas12a differ more fundamentally in how they bind the PAM and initiate R-loop formation. SpCas9 recognizes the NGG PAM in a rigid, preorganized groove through two conserved arginine–guanine clamps, and PAM binding rapidly triggers DNA melting and stable commitment after formation of a ∼10–12 bp seed R-loop. (Anders et al., 2014; Jiang et al., 2015; Sternberg et al., 2014). In contrast, Cas12a recognizes the TTTV PAM through a flexible, induced-fit interface spanning the PI and WED domains, and requires a longer, stepwise, and reversible R-loop propagation (∼15–20 bp) before stable locking and cleavage (Yamano et al., 2016; Stella et al., 2017; Strohkendl et al., 2018; Singh et al., 2018). Consistent with this, our results show that wild-type AsCas12a and LbCas12a perform less favorably than wtSpCas9 on nucleosome-bound targets, whereas Cas12a Ultra variants with enhanced PAM binding partially overcome this barrier (Figure 5; Supplementary Figures S5–S11). Together, these results suggest that although Cas12a experiences less direct steric hindrance, its weaker and slower target engagement makes it less competitive with nucleosomes than SpCas9 for occluded targets.

Having both evoSpCas9 and HIFIv1 ranked as the top editors by both AUROC and AUPRC demonstrates their strong overall performance in cleaving desired targets while maintaining a low tolerance for potential off-targets. When the target is known to be exposed, evoSpCas9’s specificity makes it the best editor, but its overall activity, even at exposed sites, is very low. However, if the target might be obscured, HIFIv1 will be the best editor to compete for access. Future high-fidelity nucleases could be engineered to enhance editing in both naked DNA and chromatin by incorporating modifications that reduce mismatch tolerance and increase the affinity of the PAM-interfacing domain for binding to the PAM.

Gemini-seq is an *in vitro* assay that enables the rigorous comparison of Cas editors, but does not examine all factors that affect gene editing in cells. Gemini-seq and other *in vitro* assays use a fixed concentration of each Cas RNP for each target. *In vivo*, CRISPR-Cas editing must contend with delivery inefficiencies in the target cell line and with RNP degradation by cellular machinery, such as proteases and nucleases (Liu et al., 2017; Glass et al., 2018). Cas editing *in vitro* is measured by DNA cleavage, but this is only the first step for *in vivo* editing. For the desired outcome in gene editing, the cleaved DNA sequence must be erroneously repaired by cellular machinery (Xue and Greene, 2021). This further complicates the process, as potential repair mechanisms differ in their rates of error incorporation or indel formation, and the repair pathway performed is influenced by the cell cycle, further increasing potential variation in editing efficiency beyond the Cas RNP cleavage (Zaboikin et al., 2017; Mao et al., 2008). If the repair does not incorporate an error, the sequence will still be targeted for Cas editing, making editor turnover critical and increasing the value of Cas RNP residence time in the cell while contending with depletion due to degradation or cell division (Richardson et al., 2016; Kim et al., 2014). Accessing our top-performing Cas editors *in vivo* will further elucidate their performance in the more complex cellular context.

## Materials and methods

### Nucleosome library design to assess multiple Cas species

We created a dual-target sequence compatible with both Cas9 and Cas12a for use in the GEMiNI-seq assay. This 26-nt sequence incorporates the Cas9 “NGG” and Cas12a “TTTV” PAMs in sequence next to a 20-nt target sequence (Figure 1A). These target sequences were positioned at every base position across the NPSs of Widom-601, 5S, and MMTV-NucA (Yu and Buck, 2020). The NPSs were selected based on their known high affinity for the DNA sequence to wrap around a histone octamer, forming a nucleosome, and their established use *in vitro*. The Widom-601, a well-characterized artificial NPS, is extensively used *in vitro* due to its strong positioning, resulting in a precisely defined structure (Yu and Buck, 2020; Chen et al., 2017; Lowary and Widom, 1998). The 5S and MMTV NPSs are derived from nucleosomes with strong *in vivo* positioning, providing a more biologically relevant structure (Richard-Foy and Hager, 1987; Hayes and Wolffe, 1993; Dong et al., 1990; Chen et al., 2014). Each positioning of the target sequence replaces the original nucleosome nucleotide sequence, thus generating a unique sequence in the nucleosome library. Replacing base-pair sequences in NPSs has been shown not to affect nucleosome positioning (Tsompana et al., 2025; Wilson et al., 2025). For this assay, we used the target sequence with the PAM positioned both 5′ and 3′ of the 20-nt sequence, giving both conformations of the target and PAM relative to the nucleosome (Figure 1B).

### GEMiNI-seq

The protocol for GEMiNI-seq is performed as previously described, with an expanded scope to test multiple Cas editors at varying concentrations (Handelmann et al., 2023). The wtSpCas9, wtLbCas12a, and wtAsCas12a were from New England Biolabs (NEB); SpCas9 HIFIv1 and SpCas9 HIFIv2 were purchased from Thermofisher; LbCas12a Ultra and AsCas12a Ultra were from Integrated DNA Technologies (IDT). An evoSpCas9 bacterial expression plasmid was generated via mutagenesis (M495V, Y515N, K526E, R661Q) of a wild-type *S. pyogenes* Cas9 plasmid containing an N-terminal His_6_-tag, an MBP-tag, and a TEV protease cleavage site. The evoSpCas9 plasmid was transformed into BL21-Gold (DE3) competent cells (Agilent) and cultured in lysogeny broth (LB) at 37 °C to an OD_600_ of 0.6–0.8. Cells were then induced for protein expression with 0.5 mM IPTG, incubated for 16–18 h at 18 °C, and harvested by centrifugation.

To purify evoSpCas9, cells were resuspended in a buffer of 20 mM HEPES, 300 mM potassium chloride, and 10 mM imidazole at pH 8.0 with an EDTA-free protease inhibitor cocktail tablet (Sigma Aldrich) and lysed by sonication. Cell debris was removed by centrifugation, and the supernatant was applied to a 5 mL Ni-NTA affinity column (Qiagen) pre-equilibrated in lysis buffer. The column was washed with lysis buffer, and the protein was eluted with a buffer of 20 mM HEPES, 300 mM potassium chloride, and 220 mM imidazole at pH 8.0. The salt concentration of the sample was adjusted to 250 mM potassium chloride, and then the sample was applied to a 10 mL heparin column (Bio-Rad) pre-equilibrated in 20 mM HEPES, 250 mM potassium chloride, and 1 mM DTT at pH 7.5. The column was washed with the equilibration buffer, and the protein was gradient eluted with 20 mM HEPES, 1.5 M potassium chloride, and 1 mM DTT at pH 7.5. The N-terminal His_6_-tag and MBP-tag were cleaved by TEV protease overnight at 4 °C while being dialyzed into lysis buffer and then removed by Ni-NTA chromatography. The sample was further purified on a HiLoad 26/600 Superdex 200 size exclusion column (Cytiva) in a buffer of 20 mM HEPES and 200 mM potassium chloride at pH 7.8. evoSpCas9 samples were dialyzed into 20 mM HEPES, 250 mM potassium chloride, and 1 mM DTT at pH 7.5, flash frozen, and stored at −80 °C.

All Cas nucleases were diluted and stored in the NEB dilution buffer for Cas9 variants and the Cas12a diluent for Cas12a variants. The sgRNA sequences for Cas9 were designed to target the DNA sequences tiled throughout the nucleosomal structure. The desired 20-nt target was flanked by the T7 promoter sequence and the 14-nt start for the RNA scaffold, following New England Biolabs (NEB) protocol for target-specific oligo design. The DNA oligonucleotides were ordered from Integrated DNA Technologies, and the sgRNAs were produced using the NEB EnGen sgRNA Synthesis Kit (NEB #E3322). To ensure proper formation, the sgRNAs were run on a 10% denaturing TBE–urea gel, and each concentration was quantified. Each sample was diluted with nuclease-free water to a 300 nM working concentration. The crRNA sequences for Cas12a were designed to target the DNA sequences tiled throughout the nucleosome. The desired 20-nt target was designed using the IDT CRISPR-Cas12a (Cpf1) crRNA Oligo Entry system and ordered from IDT. The dry crRNA was suspended in nuclease-free water.

Nucleosome libraries containing 7,500 230-bp nucleosome sequences were designed and acquired from Agilent as custom oligo libraries. The DNA sequences were amplified by PCR, column-purified using Qiagen QIAquick PCR Purification Kit, and quantified. Nucleosomes were formed from H2A/H2B dimer (160 pmol) and H3/H4 tetramer (80 pmol) from Epicypher, combined with 52.5 pmol of DNA, 1.68 M of sodium chloride, and 1 µM of dithiothreitol diluted to 100 µL with TE (pH 8.0). The reaction was incubated at room temperature for 30 min and then transferred into a Slide-A-Lyzer MINI Dialysis Unit (10,000 MWCO, Thermo Scientific No. 69750). The dialysis unit was then sequentially placed on top of 1.2 mL of 1.0, 0.8, and 0.6 M sodium chloride for 2 h each at 4 °C. The dialysis unit was then placed on top of 1.2 mL of TE (pH 8.0) overnight at 4 °C. The nucleosome sample was collected and then purified with a sucrose gradient. Fresh 20% and 7% sucrose solutions were created, and then a 7%–20% sucrose gradient was prepared using a gradient mixer. The nucleosome sample was loaded onto the sucrose gradient and then centrifuged on a SW41 rotor at 35,000 rpm for 18 h at 4 °C. The sucrose gradient was fractionated, and a portion of each fraction was run on a 4% native polyacrylamide gel to determine which fraction contained properly formed nucleosomes. Nucleosome fractions were then concentrated and washed with TE (pH 8.0) in Amicon Ultra 0.5 mL 30k filters. The concentration of the nucleosome sample was determined using a Qubit Flex fluorometer (Carpenter et al., 2023).

For testing varying RNP concentrations, the CRISPR/Cas nucleases and corresponding gRNA were incubated in a 1:1 ratio in NEBuffer 3.1 for Cas9 and NEBuffer r2.1 for Cas12a at room temperature for 10 min. Varying RNP concentrations of 120nM, 60nM, 30nM, and 15 nM were added to nucleosomes (3 nM), digested at 37 °C for 30 min, and then placed on ice. To test the impact of the Cas concentration on nucleosome cleavage, we performed 3 biological replicates for wtSpCas9, wtAsCas12a, wtLbCas12a, and LbCas12a ULTRA, and a single replicate for evoSpCas9, AsCas12a ULTRA, HIFIv1, and HIFIv2 (Figure 3; Supplementary Figures S1–S7). To test the nuclease’s ability to tolerate multiple mismatches, a single 30 nM concentration of Cas nuclease was incubated with guide RNA of 60 nM added to nucleosomes (3 nM), digested at 37 °C for 30 min, and then placed on ice. We performed 5 biological replicates for testing the mismatch tolerance of wtLbCas12a, wtAsCas12a, LbCas12a ULTRA, AsCas12a ULTRA, wtSpCas9, evoSpCas9, and HIFIv1 (Figures 2, 4–6; Supplementary Figures S8–S23). For all digests, proteinase K (20 µg) was added, and the reaction was incubated at 55 °C for 90 min. The DNA was purified from the reaction and concentrated using a Qiagen MiniElute Purification Kit. An undigested nucleosome library control was processed concurrently as a negative control. Four non-target spike-in sequences were designed and pooled to cover a range of three orders of magnitude, spanning the individual sequence concentrations within the nucleosome library. Illumina sequencing libraries were generated using two-step PCR, with 12 cycles of amplification for the first step using four sets of primers designed to offset sequence reads and dephase the libraries during Illumina sequencing (Handelmann et al., 2023). The second eight-cycle PCR barcodes the individual samples using Nextera Index primers for identification. The concentration of each sample was determined using the Invitrogen Quant-iT dsDNA Assay Kit, and equal amounts of each sample were pooled and sequenced at the UB Genomics and Bioinformatics Core.

### GEMiNI-seq analysis

The Illumina sequence reads were processed with a pipeline of applications to refine and identify the sequences present in the sample pool. The low-quality results at the 3′end of the Illumina reads were removed by the Cutadapt tool (v3.5), using a quality cutoff (−q) of 30 (26). The forward and reverse reads were merged using the Vsearch (v2.8.1) --fastq_mergepairs, only merging sequences with at least 20 overlapping nucleotides (--fastq_minovlen 20) and only allowing a maximum of two mismatches between merged sequences (--fastq_maxdiffs 2) (Rognes et al., 2016). The primer sequences present at the end of the reads were removed by Cutadapt. Any sequences >220 or <174 nt were removed, using the Cutadapt--maximum-length and--minimum-length functions, respectively. The reads were converted from fastq to fasta format using the FASTX-Toolkit (v0.0.14) FASTQ-to-FASTA converter function (http://hannonlab.cshl.edu/fastx_toolkit/index.html) before using Vsearch to identify each sequence in the nucleosome library (Rognes et al., 2016). The search was performed by comparing the processed reads to the fasta formatted nucleosome library sequences (--dbmatched), rejecting sequence matches that have alignment lengths <150 nt (--mincols 150) or have <98.5% similarity (--id 0.985), and only reporting the hit with the highest percentage of identity (--top hits only).

The number of reads for each sequence present in a sample was compared relative to the median value of the nonspecific background sequences, thus controlling for technical variability introduced by PCR, NGS library construction, and NGS sequencing. ‘Cas Cleavage’ was defined as

$$-\log_{2}\left(\frac{\frac{Reads\;Digested\;N}{Reads\;Digested\;Con}}{\frac{Reads\;Undigested\;N}{Reads\;Undigested\;Con}}\right)$$

where *Reads Digested N* was the number of uncleaved reads for a given nucleosomal target sequence in the sample pool after Cas9 cleavage, *Reads Digested Con* was the number of reads for the median value of the nonspecific sequences in the sample pool after Cas9 cleavage, *Reads Undigested N* was the number of reads for a given nucleosomal target sequence in the undigested sample pool and *Reads Undigested Con* was the median value of the nonspecific sequences in the undigested sample pool. The Z-score then normalized each sample with the mean and standard deviation defined from the nonspecific background sequences. Each target in the library was ranked within the sample based on the amount of cleavage. The targets were labeled as containing a Cas target or not, and then Specificity and Sensitivity were calculated iteratively based on the ratios of True Positive, True Negative, False Positive, and False Negative.

$$Specificity=\frac{TN}{TN+FP}$$

$$Sensitivity=\frac{TP\;}{TP+FN}$$

The Receiver Operating Characteristic (ROC) and Precision-Recall Curve (PRC) for each Cas editor at each concentration were then calculated. Area under the ROC (AUROC) and Area under the PRC (AUPRC) were calculated in R using the trapezoidal rule. The cumulative frequency for each mismatch was summed across the ranked library for comparison against the FPR.

The known concentrations for the spike-in sequences added to a library sample allowed for a direct quantification of the library sequences and a direct calculation of the number of molecules for each sequence within the nucleosome library. The spike-ins were used to calculate a trend line, and the counts were fit using the linear equation. The ‘Percent Cleavage’ for a given target sequence was calculated by

$$\frac{molecules\;\;undigested\;\;Con-molecules\;\;remaining\;\;N\;}{molecules\;\;undigested\;\;Con}$$

where molecules remaining N were the number of molecules remaining after cleavage, molecules undigested Con was the number of molecules in the undigested control. The percent cleavage was calculated at each RNP concentration (0nM, 15nM, 30nM, 60nM, and 120 nM), and a hyperbolic curve was fit to the data RStudio. The B_max_ and the K_C_ value for each curve were calculated using

$$\frac{Bmax\times E}{Kc+E}$$

where B_max_ was the maximum amount of Cas cleavage of the target, K_C_ was the concentration at which half of the targets were cleaved, and E was the concentration of the Cas RNP. The B_max_ and K_C_ were calculated for each target tiled throughout a nucleosome for each editor.

## Data Availability

The data presented in this study have been deposited in the NCBI BioProject database under accession number PRJNA1371373.
