# Supplementary material for: Evaluating high-fidelity CRISPR-Cas nucleases in nucleosomal contexts using a quantitative framework
Source: Front Genome Ed. 2026 Aug 14;8:1759382. doi: 10.3389/fgeed.2026.1759382 (PMC13522198; doi:10.3389/fgeed.2026.1759382)
Supplement: Supplementary file 1 [file DataSheet1.pdf]

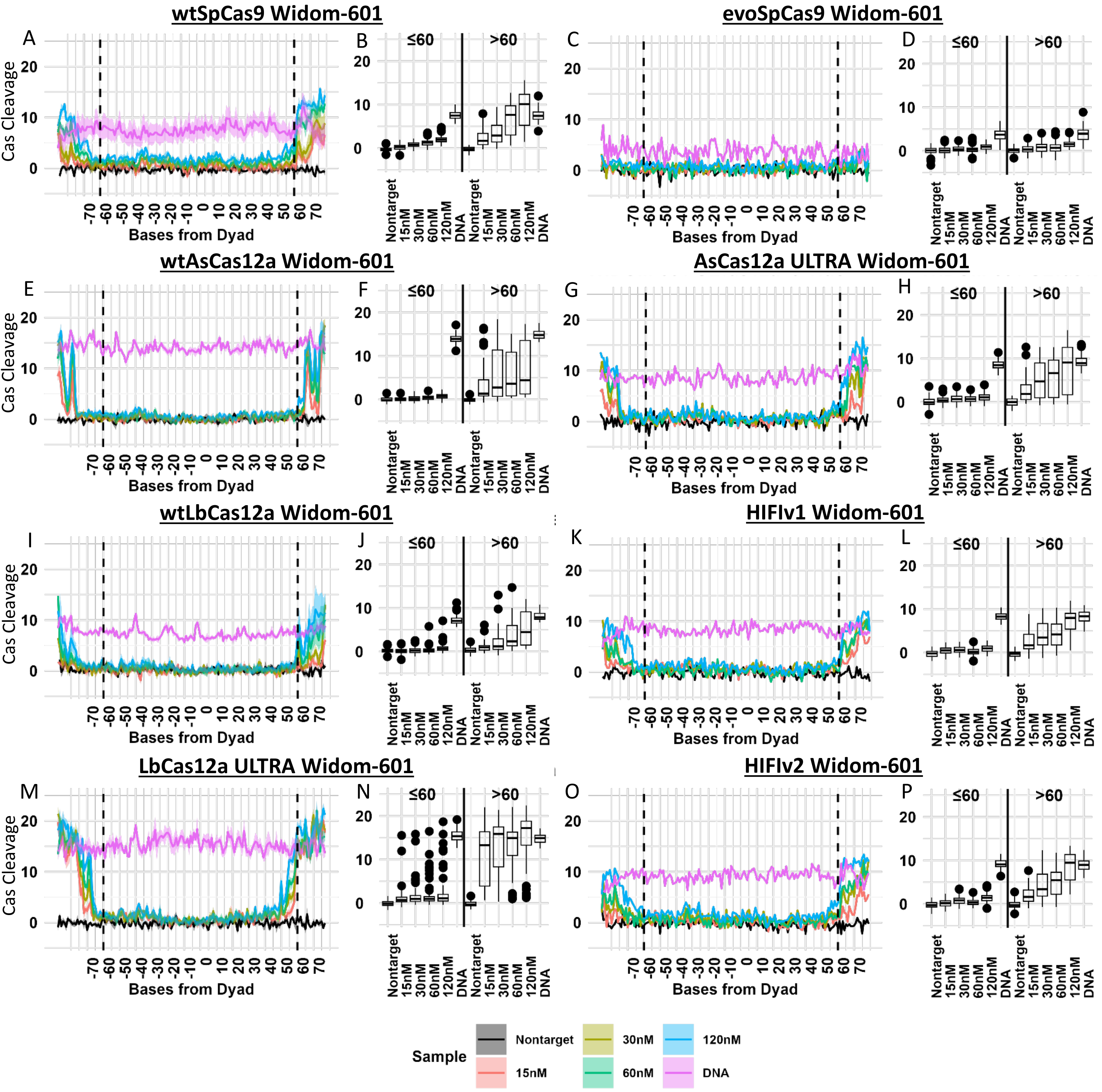

Supplemental Figure 1. The Widom-601 NPS shows a landscape of varying accessibility for different Cas editors. The digestion of the Widom-601 NPS 5' PAM at every base pair by **(A)** wtSpCas9, **(C)** evoSpCas9, **(E)** wtAsCas12a, **(G)** AsCas12a ULTRA, **(I)** wtLbCas12a, **(K)** SpCas9 High Fidelity variant 1 (HIFIv1), **(M)** LbCas12a ULTRA, and **(O)** SpCas9 High Fidelity variant 2 (HIFIv2) within a nucleosome structure at 15nM, 30nM, 60nM and 120nM concentration, in a nontarget, and in naked DNA at 120nM concentration. Black dashed lines indicate the SHL position for -6 and 6, 60 base pairs from the dyad. All digestions were performed on a 3nM nucleosome library concentration. (**B, D, F, H, J, L, N, and P**) Each Cas editor's digestion is averaged for positions within the nucleosome (SHL -6 to SHL 6) and in the linker (positions beyond SHL -6 and SHL 6) for the nontarget, the 15nM, 30nM, 60nM and 120nM Cas RNP concentrations targeting the nucleosome structure and the 120nM Cas RNP digestion on naked DNA. The ribbons within the line graphs are the standard error of the mean for the 3 biological Cas editor replicates. The digestions of wtSpCas9, wtAsCas12a, wtLbCas12a, and LbCas12a ULTRA have three biological replicates. The digestions of evoSpCas9, AsCas12a ULTRA, HIFIv1, and HIFIv2 have one biological replicate.

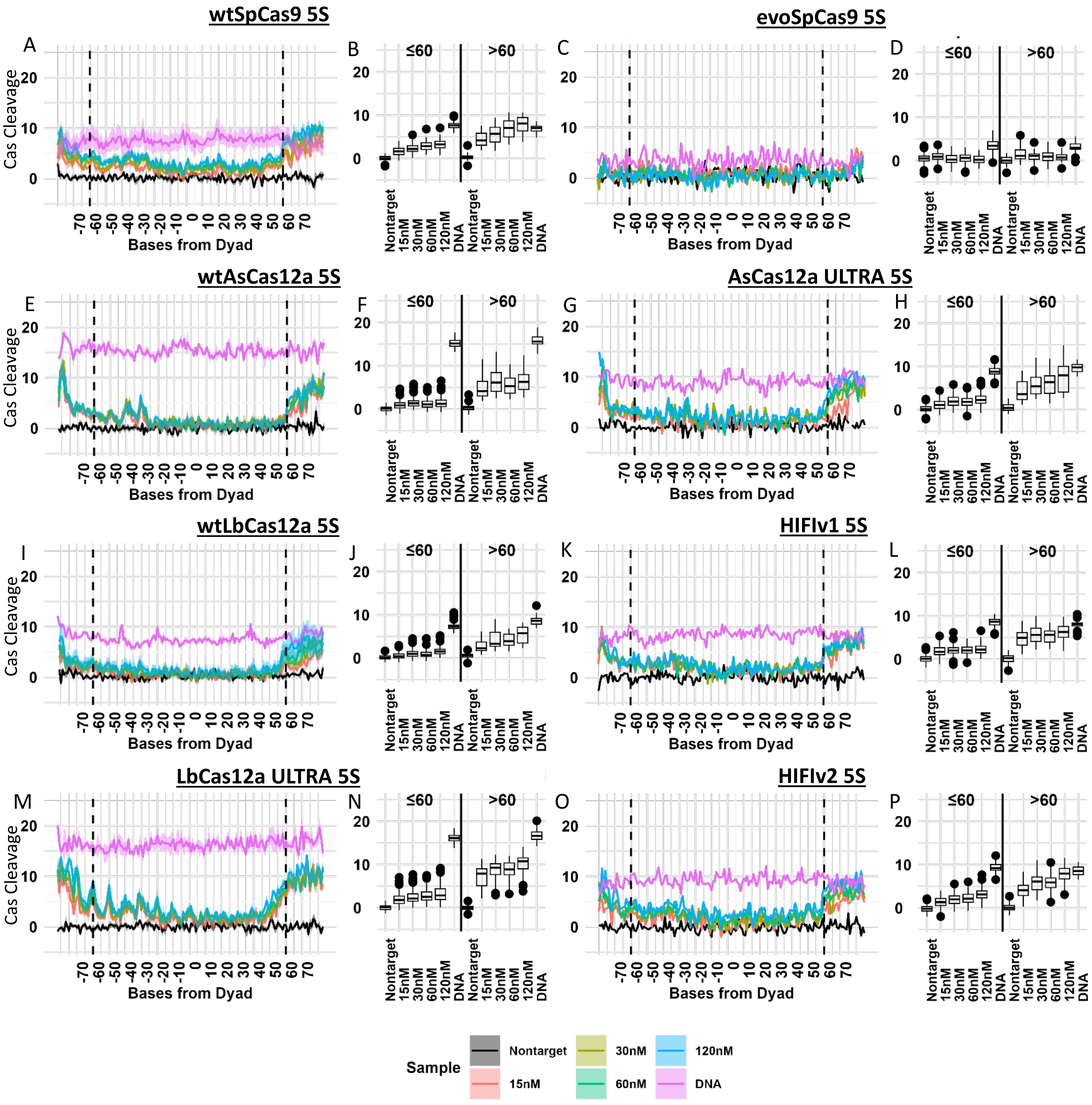

Supplemental Figure 2. Cas editors have greater target accessibility within the 5S NPS. The digestion of the 5S NPS on the 5' PAM at every base pair by **(A)** wtSpCas9, **(C)** evoSpCas9, **(E)** wtAsCas12a, **(G)** AsCas12a ULTRA, **(I)** wtLbCas12a, **(K)** HIFIv1, **(M)** LbCas12a ULTRA, and **(O)** HIFIv2 within a nucleosome structure at 15nM, 30nM, 60nM and 120nM concentration, in a nontarget, and in naked DNA at 120nM concentration. Black dashed lines indicate the SHL position for -6 and 6, 60 base pairs from the dyad. All digestions were performed on a 3nM nucleosome library concentration. (**B, D, F, H, J, L, N, and P**) Each Cas editor's digestion is averaged for positions within the nucleosome (SHL -6 to SHL 6) and in the linker (positions beyond SHL -6 and SHL 6) for the nontarget, the 15nM, 30nM, 60nM and 120nM Cas RNP concentrations targeting the nucleosome structure and the 120nM Cas RNP digestion on naked DNA. The ribbons within the line graphs are the standard error of the mean for the 3 biological Cas editor replicates. The digestions of wtSpCas9, wtAsCas12a, wtLbCas12a, and LbCas12a ULTRA have three biological replicates. The digestions of evoSpCas9, AsCas12a ULTRA, HIFIv1, and HIFIv2 have one biological replicate.

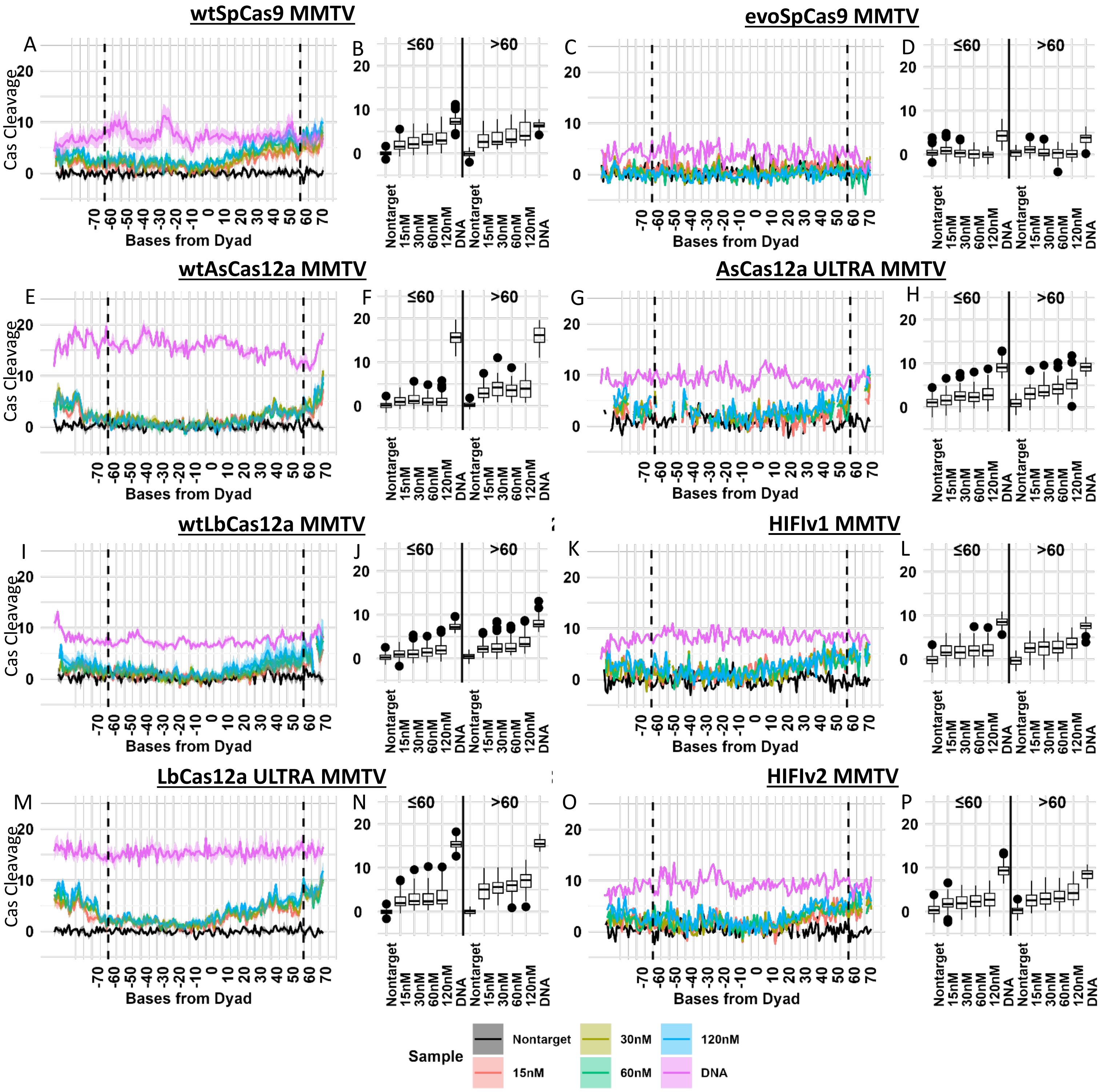

Supplemental Figure 3. The MMTV NPS landscape for Cas editors accessibility to the 5' PAM The digestion of the MMTV NPS at every base pair by **(A)** wtSpCas9, **(C)** evoSpCas9, **(E)** wtAsCas12a, **(G)** AsCas12a ULTRA, **(I)** wtLbCas12a, **(K)** SpCas9 High Fidelity variant 1 (HIFIv1), **(M)** LbCas12a ULTRA, and **(O)** SpCas9 High Fidelity variant 2 (HIFIv2) within a nucleosome structure at 15nM (pink), 30nM (yellow), 60nM (green) and 120nM concentration (blue), in a nontarget (black), and in naked DNA at 120 nM concentration(purple). Black dashed lines indicate the SHL position for -6 and 6, 60 base pairs from the dyad. All digestions were performed on a 3nM nucleosome library concentration. (**B, D, F, H, J, L, N, and P**) Each Cas editor's digestion is averaged for positions within the nucleosome (SHL -6 to SHL 6) and in the linker (positions beyond SHL -6 and SHL 6) for the nontarget, the 15nM, 30nM, 60nM and 120nM Cas RNP concentrations targeting the nucleosome structure and the 120nM Cas RNP digestion on naked DNA. The ribbons within the line graphs are the standard error of the mean for the 3 biological Cas editor replicates. The digestions of wtSpCas9, wtAsCas12a, wtLbCas12a, and LbCas12a ULTRA have three biological replicates. The digestions of evoSpCas9, AsCas12a ULTRA, HIFIv1, and HIFIv2 have one biological replicate.

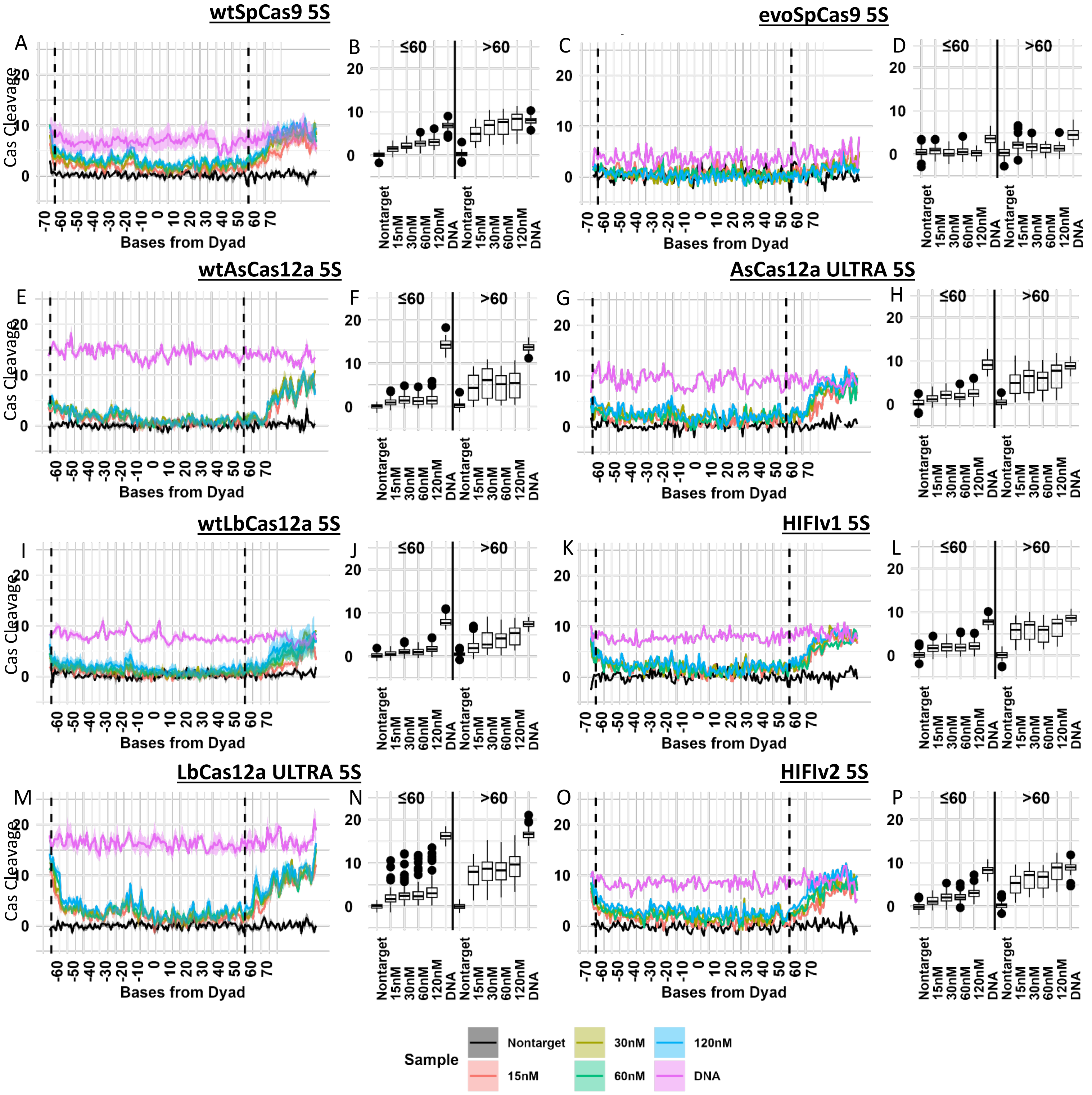

Supplemental Figure 4. The 5S NPS landscape for Cas editor accessibility to the 3' PAM.
The digestion of the 5S NPS at every base pair by **(A)** wtSpCas9, **(C)** evoSpCas9, **(E)** wtAsCas12a, **(G)** AsCas12a ULTRA, **(I)** wtLbCas12a, **(K)** SpCas9 High Fidelity variant 1 (HIFIv1), **(M)** LbCas12a ULTRA, and **(O)** SpCas9 High Fidelity variant 2 (HIFIv2) within a nucleosome structure at 15nM (pink), 30nM (yellow), 60nM (green) and 120nM concentration (blue), in a nontarget (black), and in naked DNA at 120 nM concentration(purple). Black dashed lines indicate the SHL position for -6 and 6, 60 base pairs from the dyad. All digestions were performed on a 3nM nucleosome library concentration. (**B, D, F, H, J, L, N, and P**) Each Cas editor's digestion is averaged for positions within the nucleosome (SHL -6 to SHL 6) and in the linker (positions beyond SHL -6 and SHL 6) for the nontarget, the 15nM, 30nM, 60nM and 120nM Cas RNP concentrations targeting the nucleosome structure and the 120nM Cas RNP digestion on naked DNA. The ribbons within the line graphs are the standard error of the mean for the 3 biological Cas editor replicates. The digestions of wtSpCas9, wtAsCas12a, wtLbCas12a, and LbCas12a ULTRA have three biological replicates. The digestions of evoSpCas9, AsCas12a ULTRA, HIFIv1, and HIFIv2 have one biological replicate.

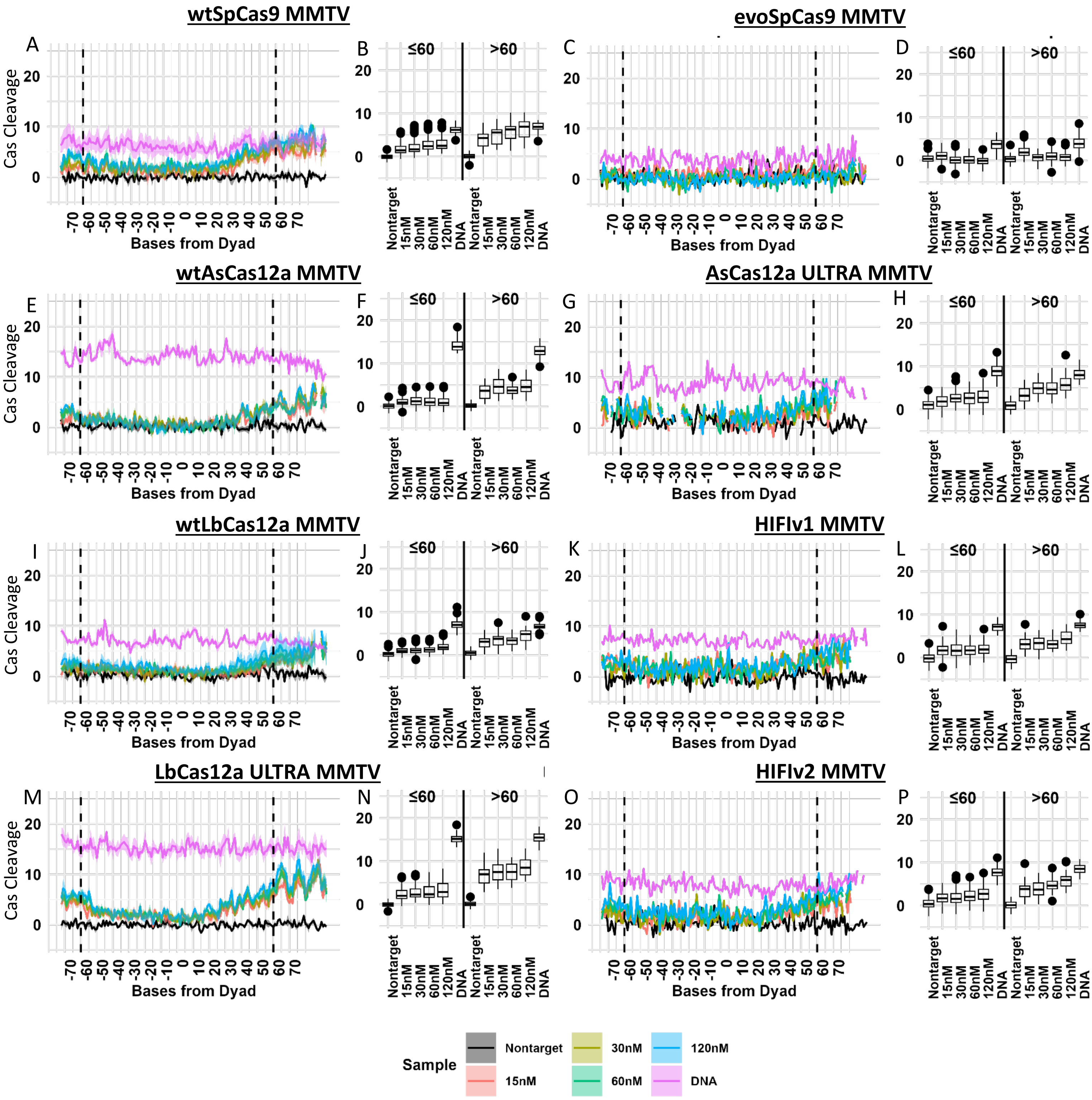

Supplemental Figure 5. The MMTV NPS landscape for Cas editors accessibility to the 3' PAM.

The digestion of the MMTV NPS at every base pair by **(A)** wtSpCas9, **(C)** evoSpCas9, **(E)** wtAsCas12a, **(G)** AsCas12a ULTRA, **(I)** wtLbCas12a, **(K)** SpCas9 High Fidelity variant 1 (HIFIv1), **(M)** LbCas12a ULTRA, and **(O)** SpCas9 High Fidelity variant 2 (HIFIv2) within a nucleosome structure at 15nM (pink), 30nM (yellow), 60nM (green) and 120nM concentration (blue), in a nontarget (black), and in naked DNA at 120 nM concentration(purple). Black dashed lines indicate the SHL position for -6 and 6, 60 base pairs from the dyad. All digestions were performed on a 3nM nucleosome library concentration. (**B, D, F, H, J, L, N, and P**) Each Cas editor's digestion is averaged for positions within the nucleosome (SHL -6 to SHL 6) and in the linker (positions beyond SHL -6 and SHL 6) for the nontarget, the 15nM, 30nM, 60nM and 120nM Cas RNP concentrations targeting the nucleosome structure and the 120nM Cas RNP digestion on naked DNA. The ribbons within the line graphs are the standard error of the mean for the 3 biological Cas editor replicates. The digestions of wtSpCas9, wtAsCas12a, wtLbCas12a, and LbCas12a ULTRA have three biological replicates. The digestions of evoSpCas9, AsCas12a ULTRA, HIFIv1, and HIFIv2 have one biological replicate.

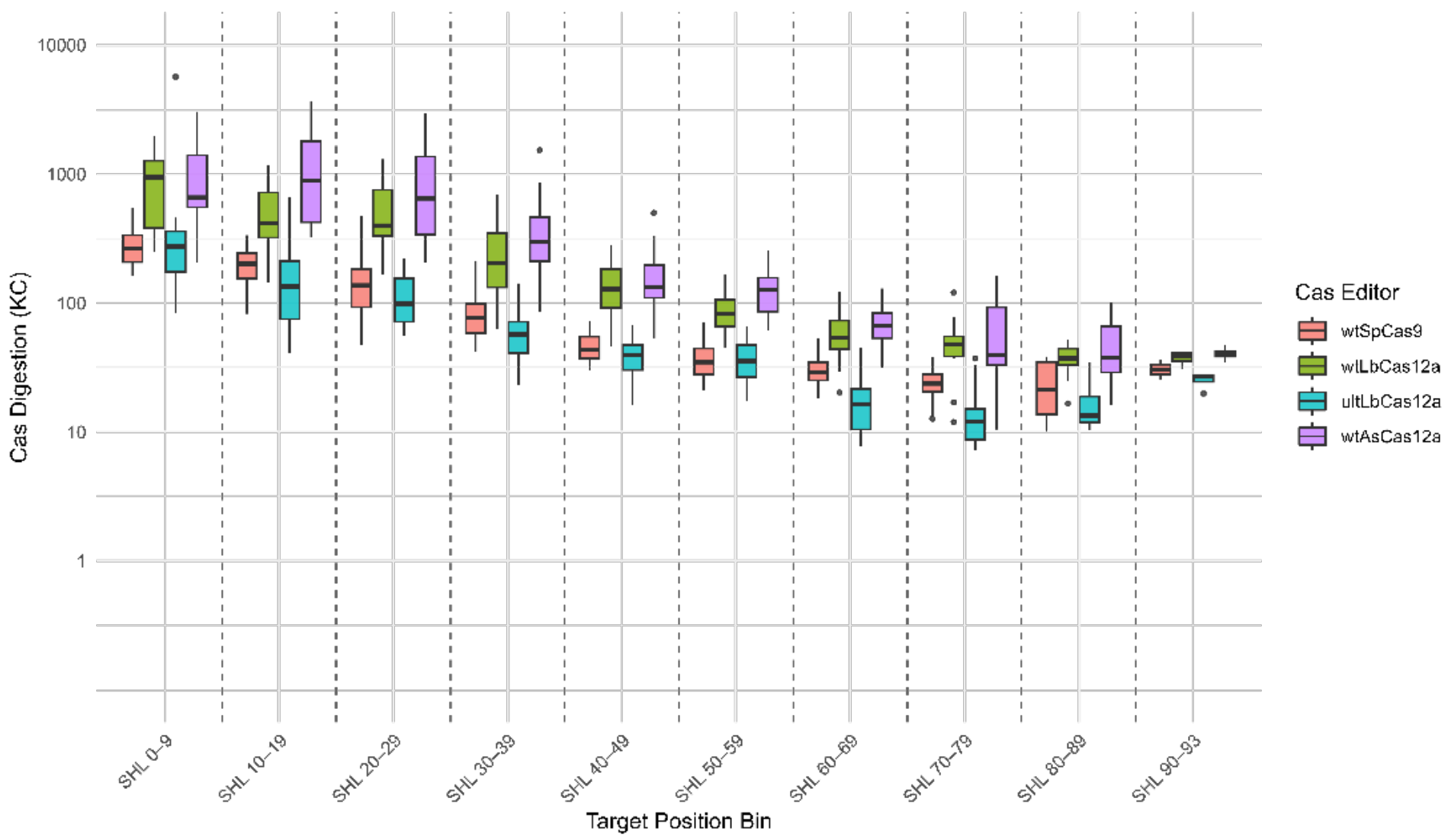

Supplemental Figure 6. The amount of Cas RNP needed to achieve target cleavage varies across the MMTV nucleosome and differs between Cas editors.

Kc values (enzyme concentration required to reach 80% cleavage, derived from nonlinear regression fits) are plotted for each Cas editor at 10 bp intervals from the dyad out into the linker for MMTV. Each section represents the Kc values for both 5' and 3' PAM-oriented targets, with Kc values grouped by SHL and plotted on a logarithmic scale to capture the wide dynamic range across nucleosomal positions. Higher Kc values reflect positions requiring more Cas RNP to achieve target cleavage, indicative of decreased digestion, whereas lower values denote more accessible targets. All Cas editors are composed of 3 biological replicates.

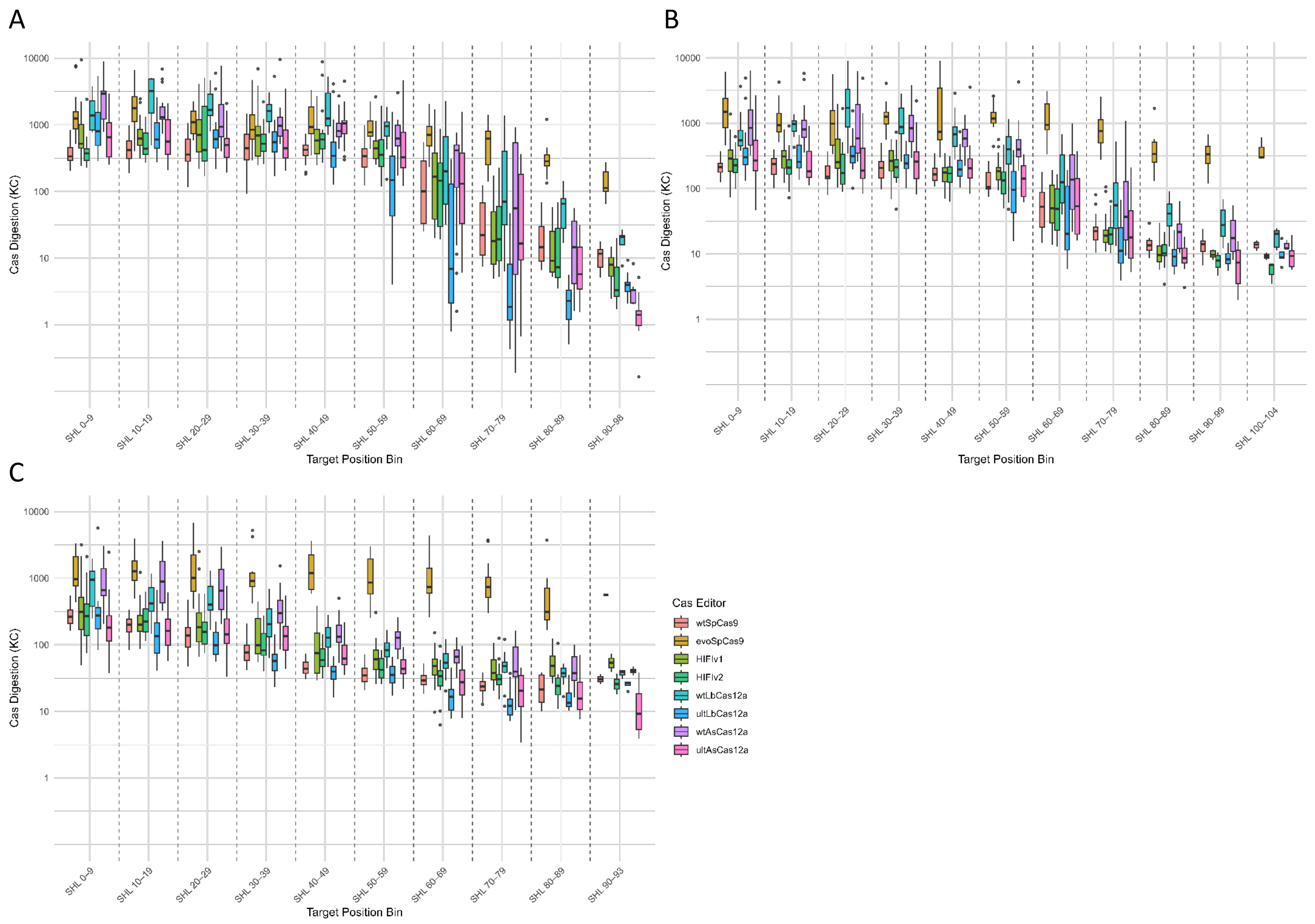

Supplemental Figure 7. The amount of Cas RNP needed to achieve target cleavage varies across nucleosome positions and differs between Cas editors.

Kc values (enzyme concentration required to reach 80% cleavage, derived from nonlinear regression fits) are plotted for each Cas editor at 10 bp intervals from the dyad out into the linker for **A)** the Widom-601, **(B)** the 5S NPS, and **(C)** MMTV. Each section represents the Kc values for both 5' and 3' PAM-oriented targets, with Kc values grouped by SHL and plotted on a logarithmic scale to capture the wide dynamic range across nucleosomal positions. Higher Kc values reflect positions requiring more Cas RNP to achieve target cleavage, indicative of decreased digestion, whereas lower values denote more accessible targets. All Cas editors are composed of 1 biological replicate.

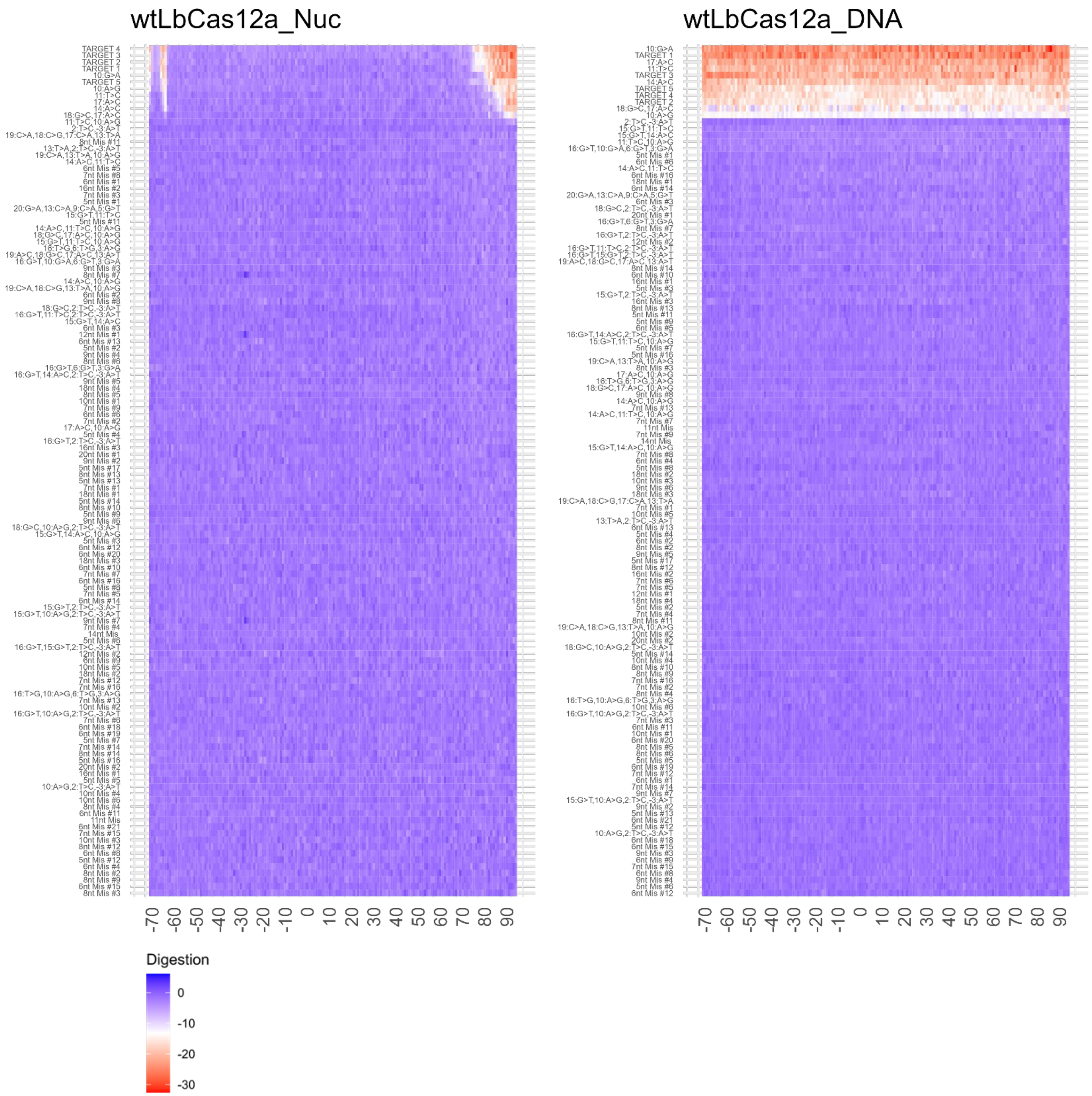

Supplemental Figure 8. Heat map of wtLbCas12a nuclease digestion on the Widom-601 NPS in both nucleosomes and naked DNA. The order of the targets on the y-axis is determined by averaging the amount of cleavage of +50 bp to +95 bp from the dyad. Amount of digestion is shown as a heatmap transitioning from blue (not digested) to red (digested).

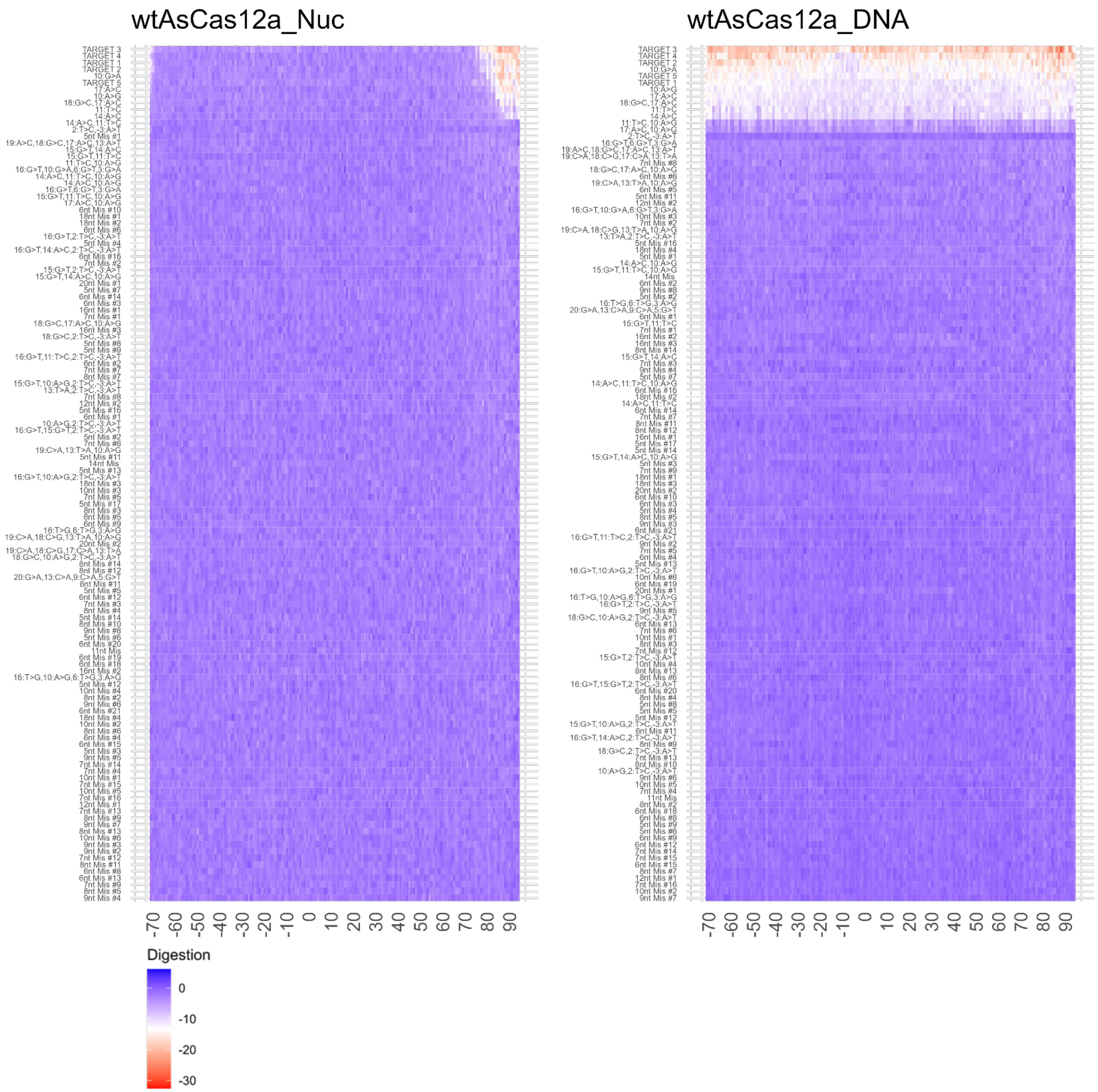

Supplemental Figure 9. Heat map of wtAsCas12a nuclease digestion on the Widom-601 NPS in both nucleosomes and naked DNA. The order of the targets on the y-axis is determined by averaging the amount of cleavage of +50 bp to +95 bp from the dyad. Amount of digestion is shown as a heatmap transitioning from blue (not digested) to red (digested).

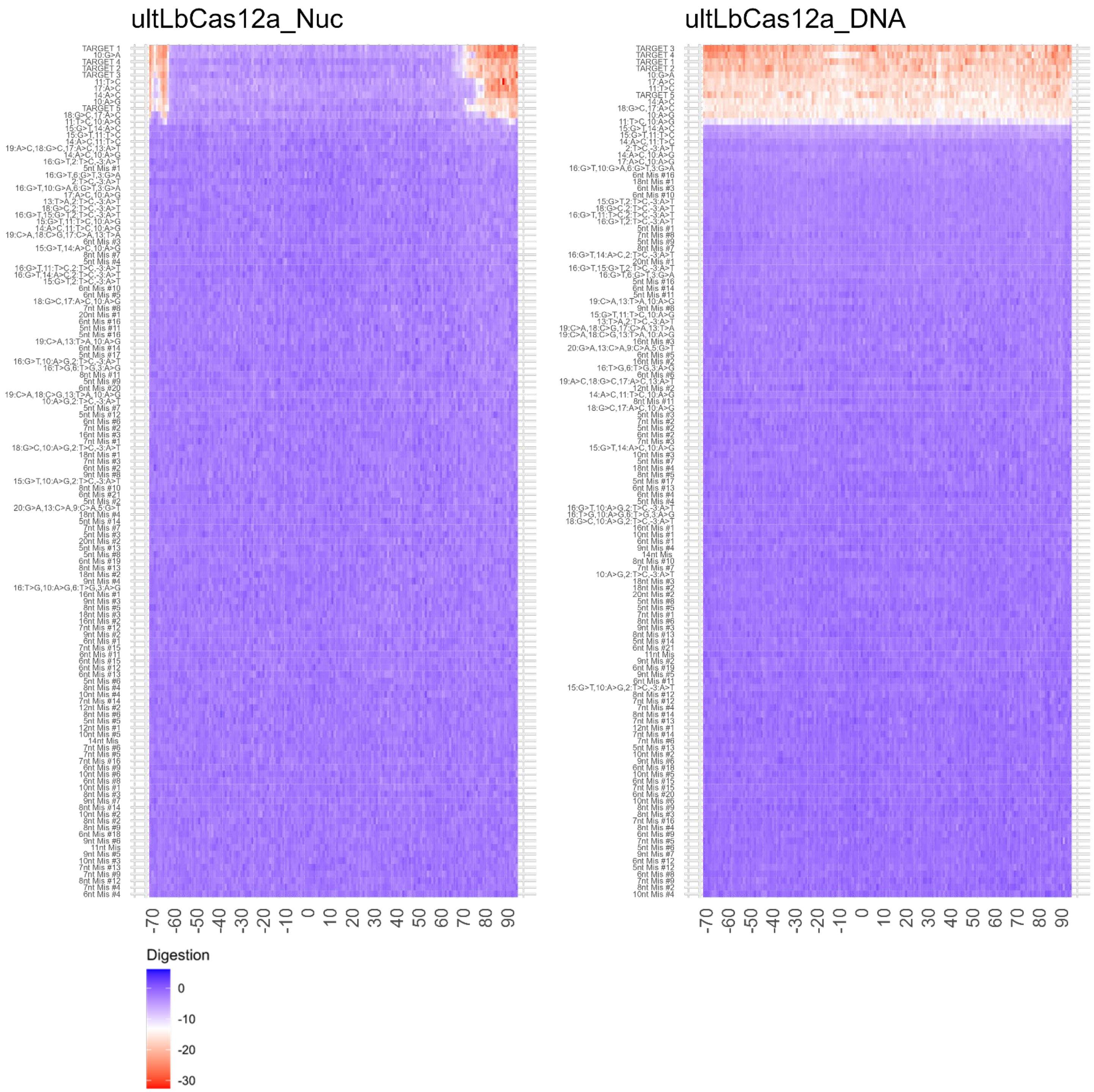

Supplemental Figure 10. Heat map of LbCas12a ULTRA nuclease digestion on the Widom-601 NPS in both nucleosomes and naked DNA. The order of the targets on the y-axis is determined by averaging the amount of cleavage of +50 bp to +95 bp from the dyad. Amount of digestion is shown as a heatmap transitioning from blue (not digested) to red (digested).

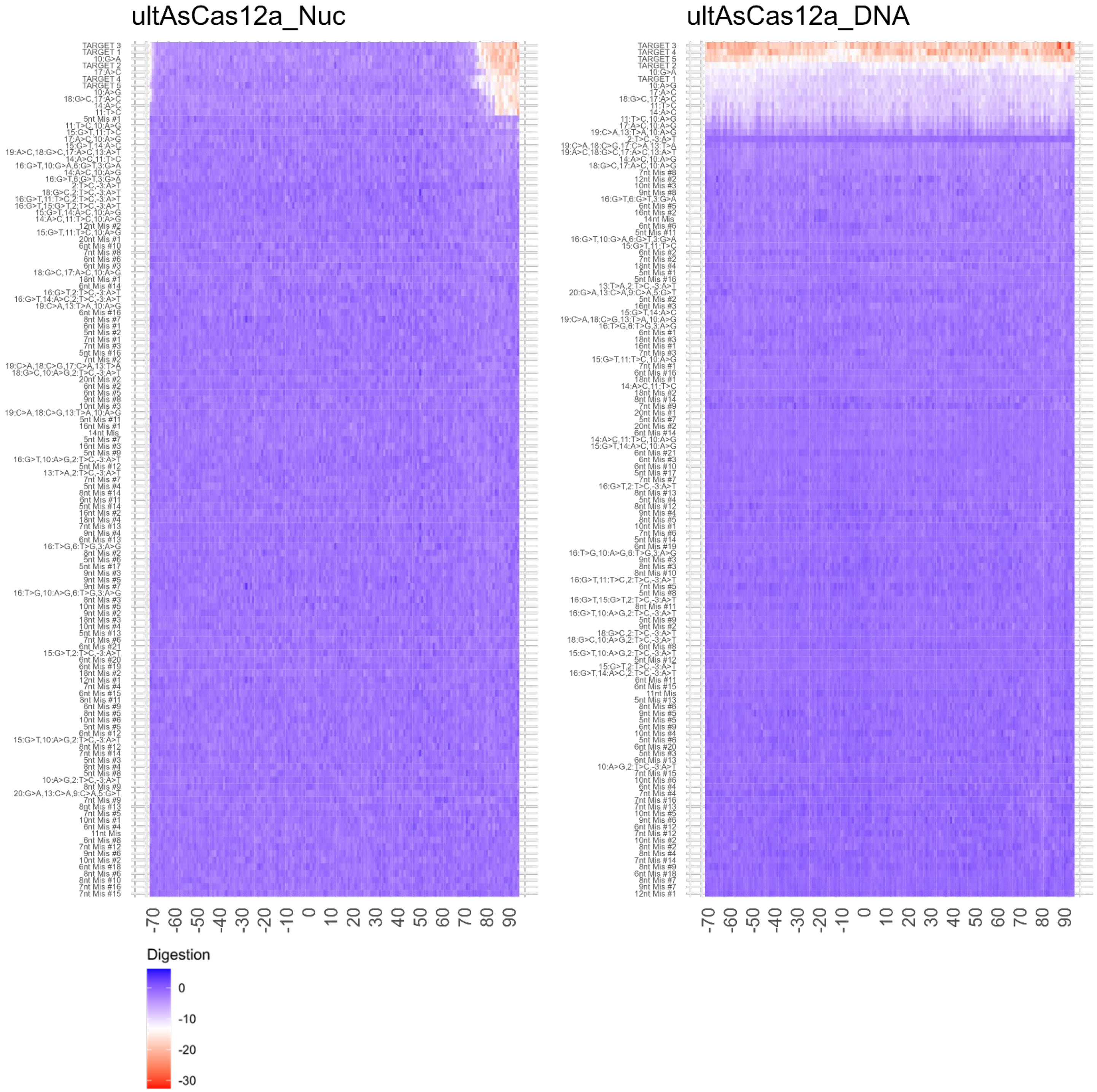

Supplemental Figure 11. Heat map of AsCas12a ULTRA nuclease digestion on the Widom-601 NPS in both nucleosomes and naked DNA. The order of the targets on the y-axis is determined by averaging the amount of cleavage of +50 bp to +95 bp from the dyad. Amount of digestion is shown as a heatmap transitioning from blue (not gigested) to red (digested).

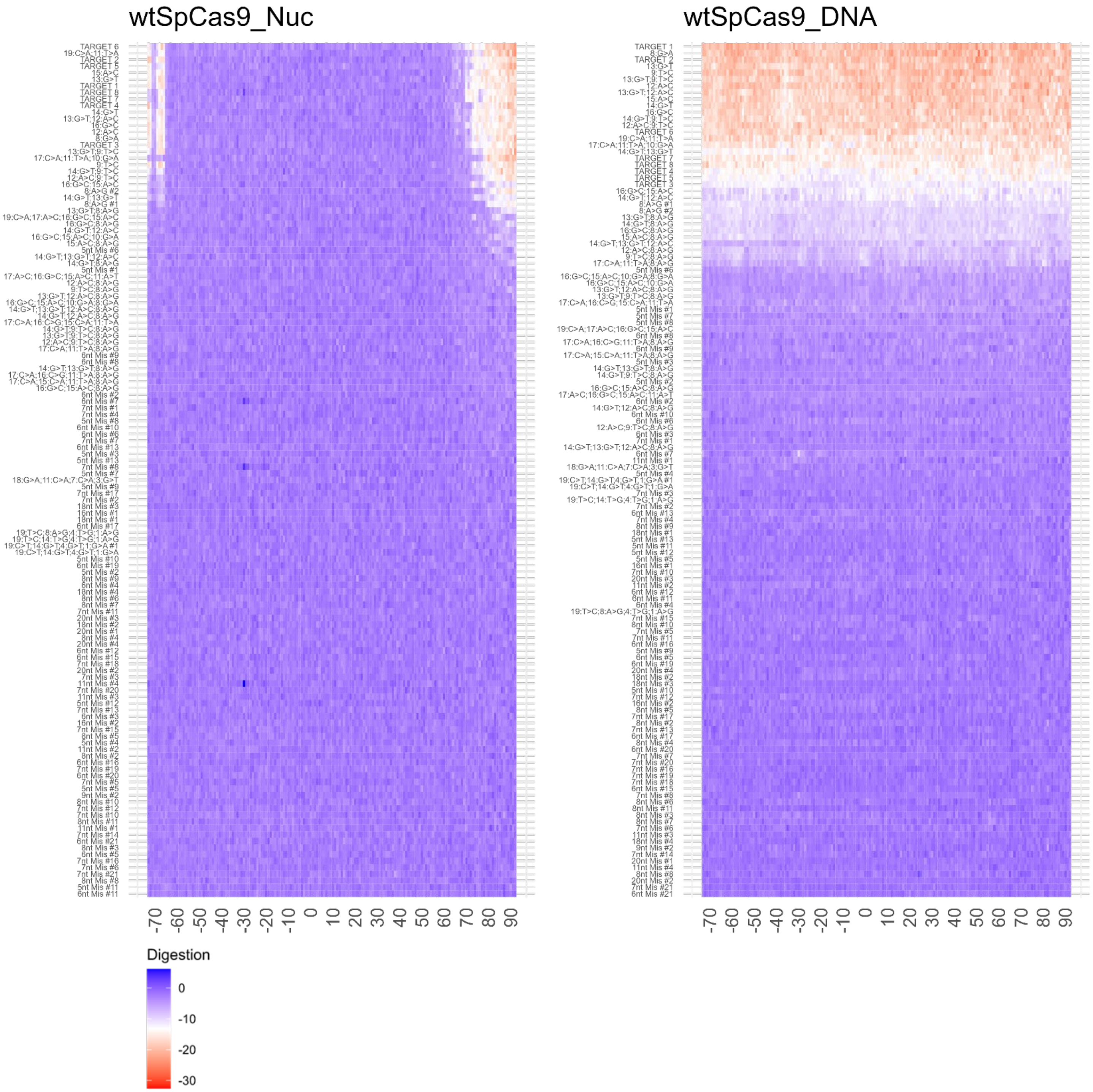

Supplemental Figure 12. Heat map of wtSpCas9 nuclease digestion on the Widom-601 NPS in both nucleosomes and naked DNA. The order of the targets on the y-axis is determined by averaging the amount of cleavage of +50 bp to +95 bp from the dyad. Amount of digestion is shown as a heatmap transitioning from blue (not digested) to red (digested).

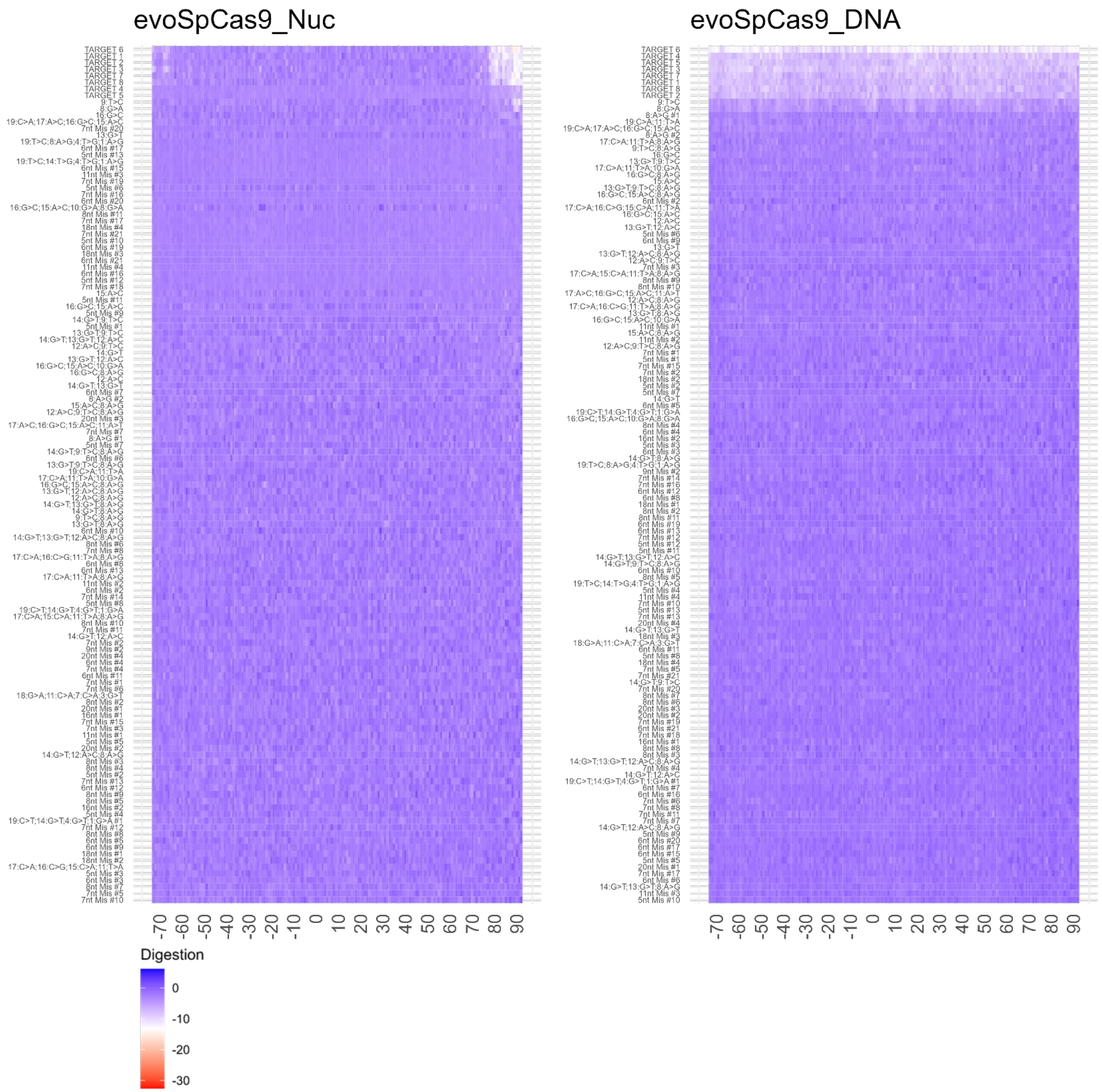

Supplemental Figure 13. Heat map of evoSpCas9 nuclease digestion on the Widom-601 NPS in both nucleosomes and naked DNA. The order of the targets on the y-axis is determined by averaging the amount of cleavage of +50 bp to +95 bp from the dyad. Amount of digestion is shown as a heatmap transitioning from blue (not digested) to red (digested).

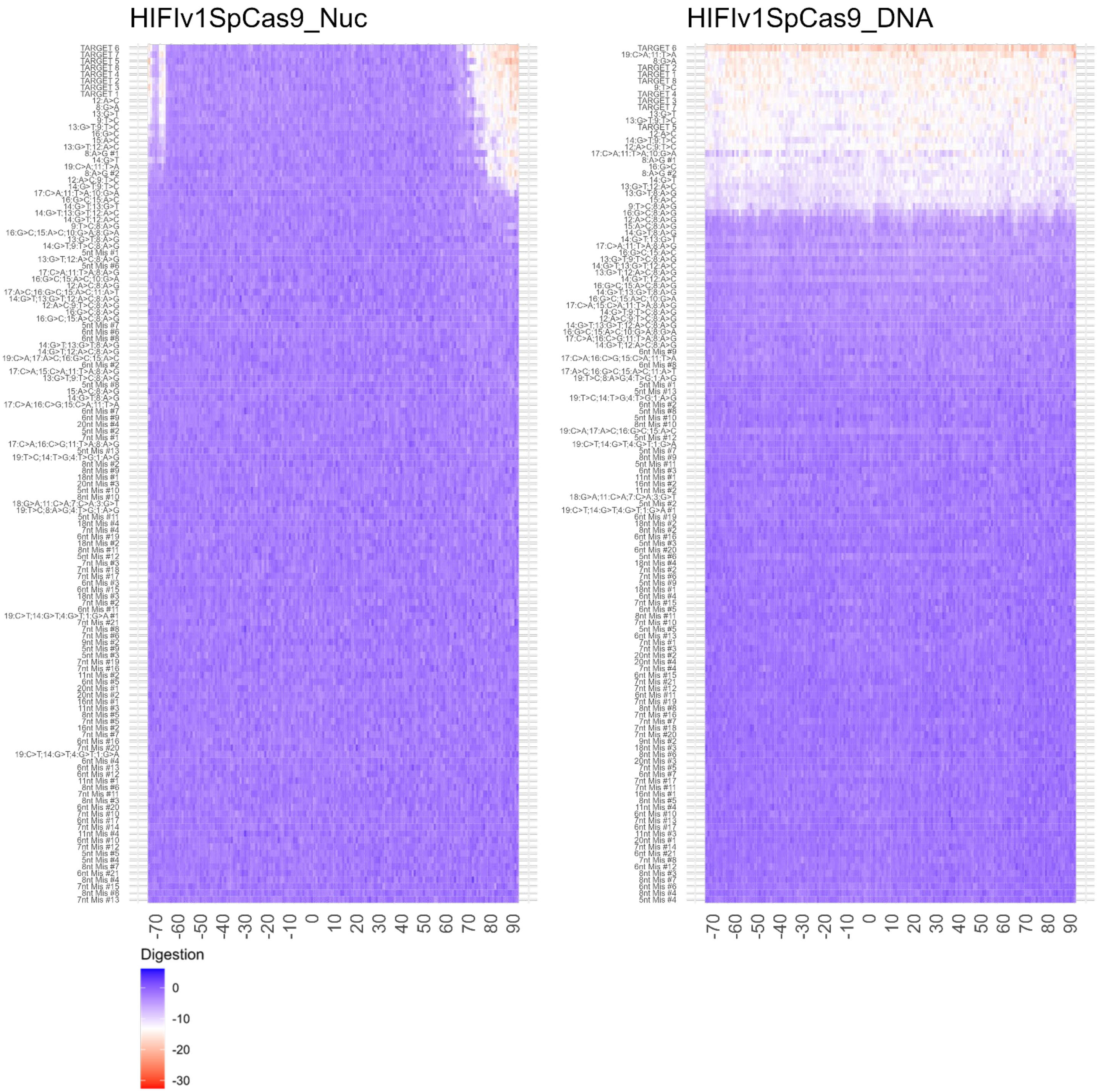

Supplemental Figure 14. Heat map of HIFIv1 nuclease digestion on the Widom-601 NPS in both nucleosomes and naked DNA. The order of the targets on the y-axis is determined by averaging the amount of cleavage of +50 bp to +95 bp from the dyad. Amount of digestion is shown as a heatmap transitioning from blue (not digested) to red (digested).

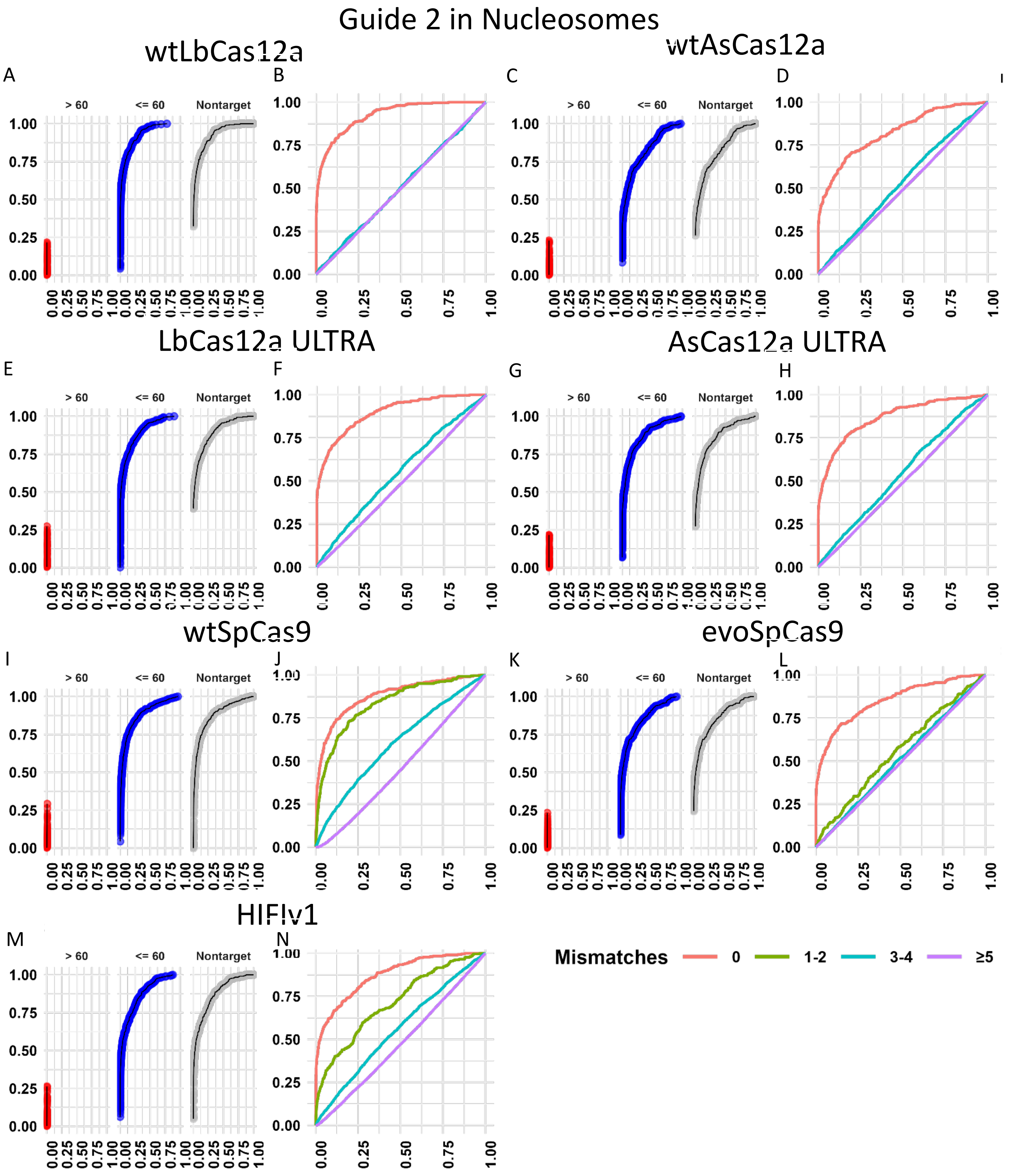

Supplemental Figure 15. Assessing the specificity and sensitivity of each Cas editor.
The 7,500 sequence library was used to evaluate the performance of each Cas editor. Sequences were ranked by cleavage efficiency, and true positive rate (TPR) versus false positive rate (FPR) was calculated to generate receiver operating characteristic (ROC) curves for **(A)** wtLbCas12a, **(C)** wtAsCas12a, **(E)** LbCas12a ULTRA, **(G)** AsCas12a ULTRA, **(I)** wtSpCas9, **(K)** evoSpCas9, and **(M)** HIFIv1. Each ROC curve is subdivided into targets within ± 60bp of the dyad, targets outside ± 60bp, and mistarget sequences. The cumulative frequency for the target sequences and varying amounts of mismatches (1-2, 3-4, and 5+) for the Cas12a variants **(B, D, F, and H)** and the Cas9 variants **(J, L, and N)**. A greater separation between target and mismatch curves indicates higher specificity. All Cas RNP digestions were at 30nM to 3nM nucleosome library.

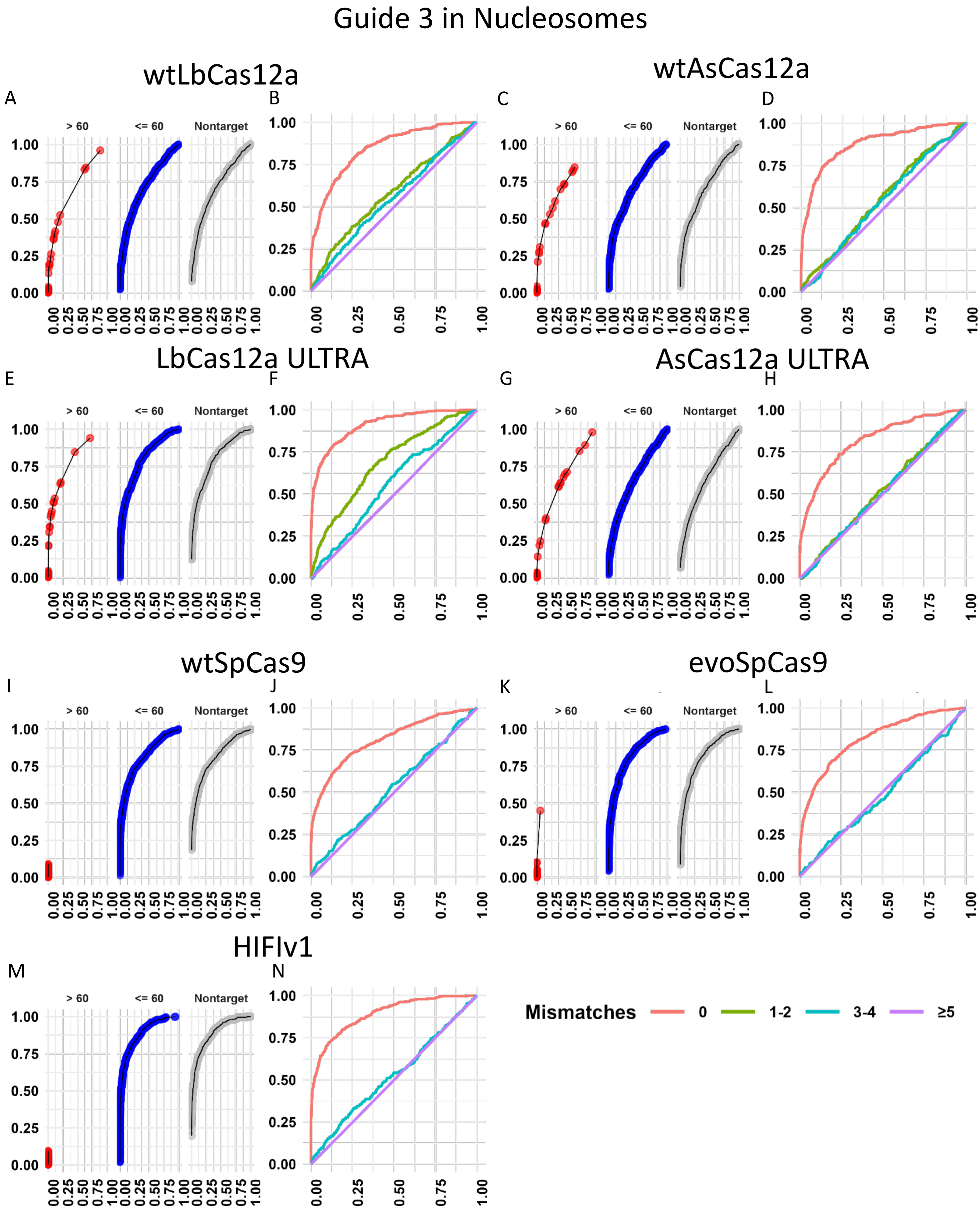

Supplemental Figure 16. Assessing the specificity and sensitivity of each Cas editor.

The 7,500 sequence library was used to evaluate the performance of each Cas editor. Sequences were ranked by cleavage efficiency, and true positive rate (TPR) versus false positive rate (FPR) was calculated to generate receiver operating characteristic (ROC) curves for **(A)** wtLbCas12a, **(C)** wtAsCas12a, **(E)** LbCas12a ULTRA, **(G)** AsCas12a ULTRA, **(I)** wtSpCas9, **(K)** evoSpCas9, and **(M)** HIFIv1. Each ROC curve is subdivided into targets within ± 60bp of the dyad, targets outside ± 60bp, and mistarget sequences. The cumulative frequency for the target sequences and varying amounts of mismatches (1-2, 3-4, and 5+) for the Cas12a variants **(B, D, F, and H)** and the Cas9 variants **(J, L, and N)**. A greater separation between target and mismatch curves indicates higher specificity. All Cas RNP digestions were at 30nM to 3nM nucleosome library.

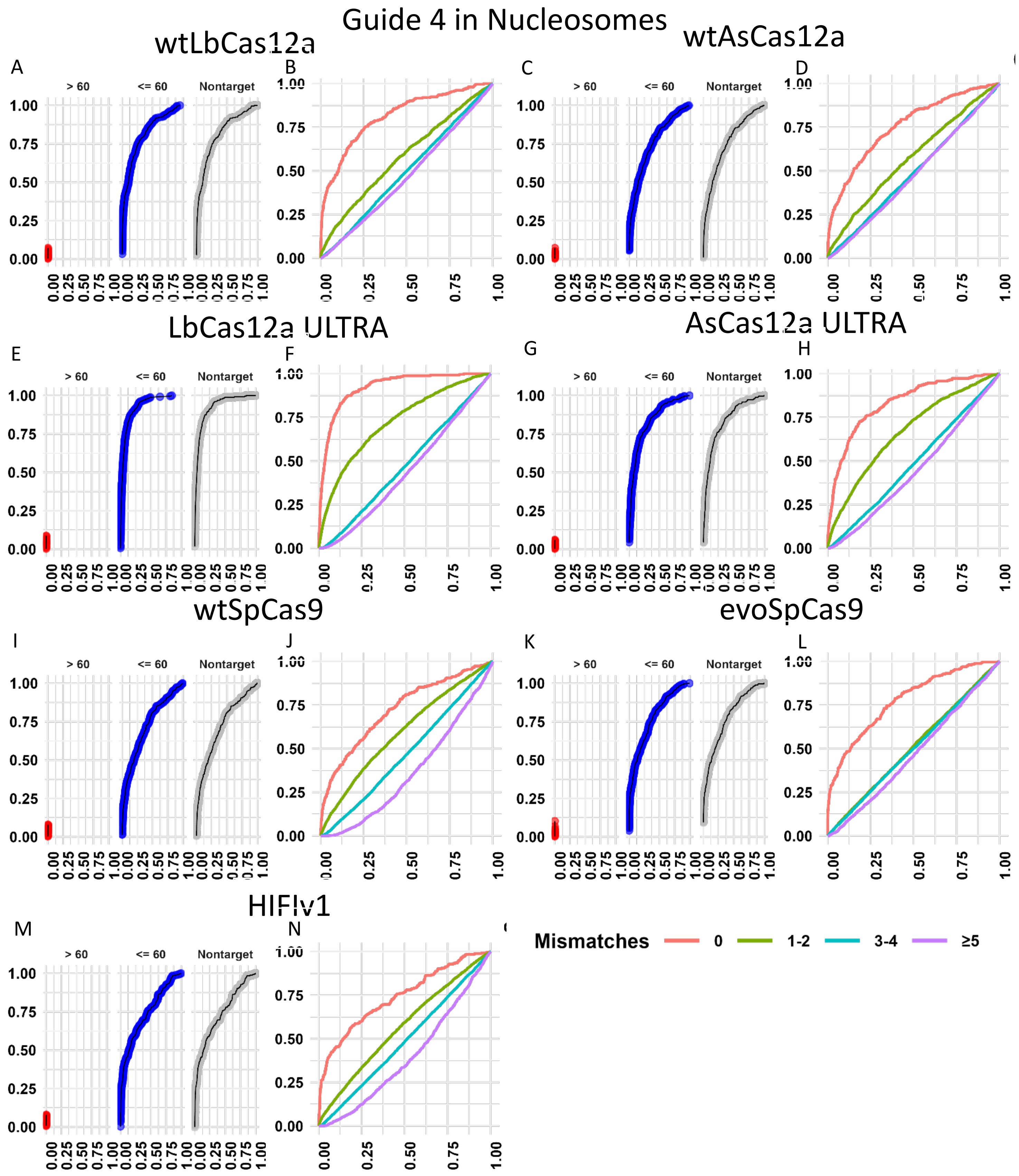

Supplemental Figure 17. Assessing the specificity and sensitivity of each Cas editor.
The 7,500 sequence library was used to evaluate the performance of each Cas editor. Sequences were ranked by cleavage efficiency, and true positive rate (TPR) versus false positive rate (FPR) was calculated to generate receiver operating characteristic (ROC) curves for **(A)** wtLbCas12a, **(C)** wtAsCas12a, **(E)** LbCas12a ULTRA, **(G)** AsCas12a ULTRA, **(I)** wtSpCas9, **(K)** evoSpCas9, and **(M)** HIFIv1. Each ROC curve is subdivided into targets within ± 60bp of the dyad, targets outside ± 60bp, and mistarget sequences. The cumulative frequency for the target sequences and varying amounts of mismatches (1-2, 3-4, and 5+) for the Cas12a variants **(B, D, F, and H)** and the Cas9 variants **(J, L, and N)**. A greater separation between target and mismatch curves indicates higher specificity. All Cas RNP digestions were at 30nM to 3nM nucleosome library.

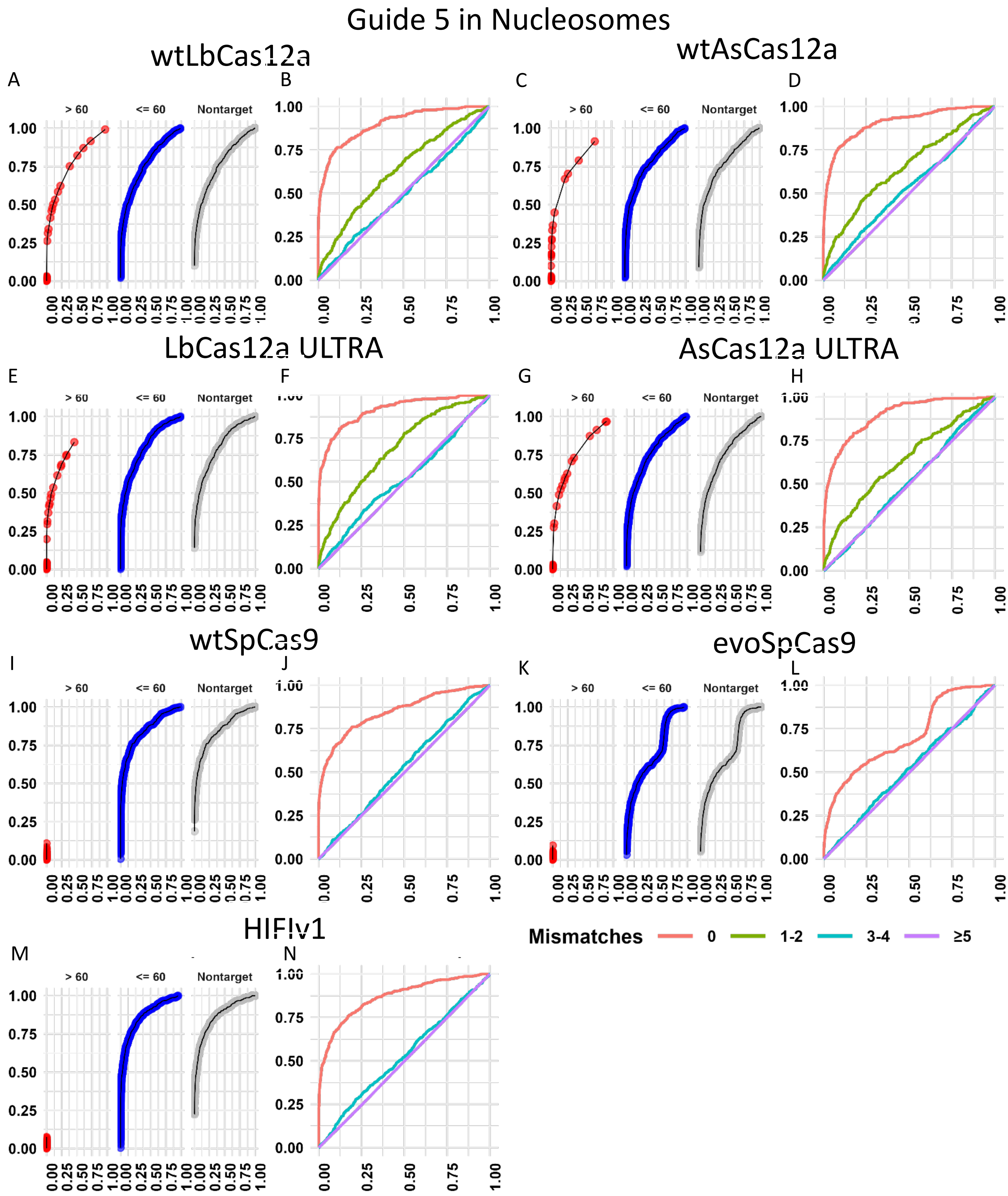

Supplemental Figure 18. Assessing the specificity and sensitivity of each Cas editor.

The 7,500 sequence library was used to evaluate the performance of each Cas editor. Sequences were ranked by cleavage efficiency, and true positive rate (TPR) versus false positive rate (FPR) was calculated to generate receiver operating characteristic (ROC) curves for **(A)** wtLbCas12a, **(C)** wtAsCas12a, **(E)** LbCas12a ULTRA, **(G)** AsCas12a ULTRA, **(I)** wtSpCas9, **(K)** evoSpCas9, and **(M)** HIFIv1. Each ROC curve is subdivided into targets within ± 60bp of the dyad, targets outside ± 60bp, and mistarget sequences. The cumulative frequency for the target sequences and varying amounts of mismatches (1-2, 3-4, and 5+) for the Cas12a variants **(B, D, F, and H)** and the Cas9 variants **(J, L, and N)**. A greater separation between target and mismatch curves indicates higher specificity. All Cas RNP digestions were at 30nM to 3nM nucleosome library.

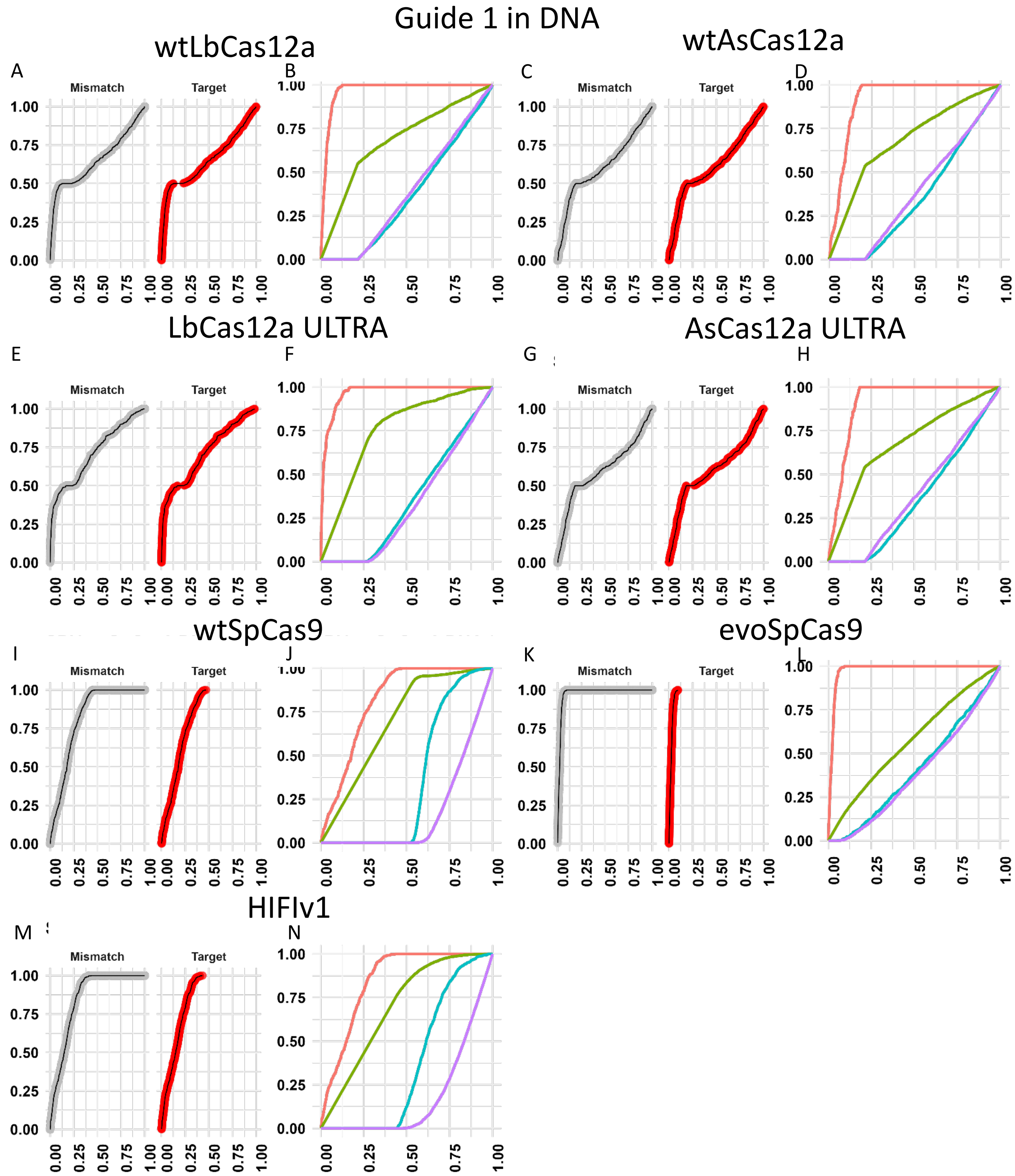

Supplemental Figure 19. Assessing the specificity and sensitivity of each Cas editor.
The 7,500 sequence library was used to evaluate the performance of each Cas editor. Sequences were ranked by cleavage efficiency, and true positive rate (TPR) versus false positive rate (FPR) was calculated to generate receiver operating characteristic (ROC) curves for **(A)** wtLbCas12a, **(C)** wtAsCas12a, **(E)** LbCas12a ULTRA, **(G)** AsCas12a ULTRA, **(I)** wtSpCas9, **(K)** evoSpCas9, and **(M)** HIFIv1. Each ROC curve is subdivided into targets within ± 60bp of the dyad, targets outside ± 60bp, and mistarget sequences. The cumulative frequency for the target sequences and varying amounts of mismatches (1-2, 3-4, and 5+) for the Cas12a variants **(B, D, F, and H)** and the Cas9 variants **(J, L, and N)**. A greater separation between target and mismatch curves indicates higher specificity. All Cas RNP digestions were at 30nM to 3nM nucleosome library.

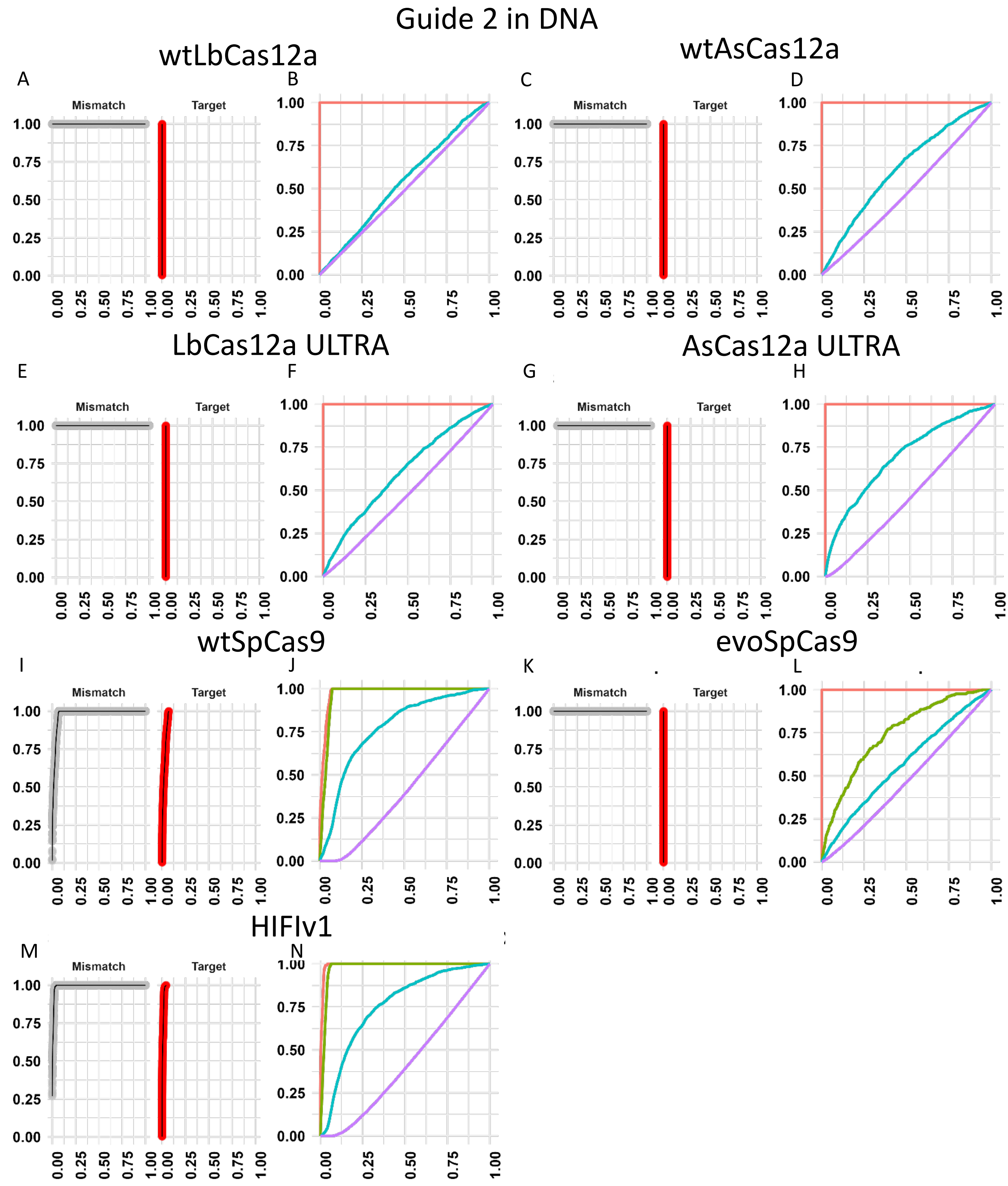

Supplemental Figure 20. Assessing the specificity and sensitivity of each Cas editor.
The 7,500 sequence library was used to evaluate the performance of each Cas editor. Sequences were ranked by cleavage efficiency, and true positive rate (TPR) versus false positive rate (FPR) was calculated to generate receiver operating characteristic (ROC) curves for **(A)** wtLbCas12a, **(C)** wtAsCas12a, **(E)** LbCas12a ULTRA, **(G)** AsCas12a ULTRA, **(I)** wtSpCas9, **(K)** evoSpCas9, and **(M)** HIFIv1. Each ROC curve is subdivided into targets within ± 60bp of the dyad, targets outside ± 60bp, and mistarget sequences. The cumulative frequency for the target sequences and varying amounts of mismatches (1-2, 3-4, and 5+) for the Cas12a variants **(B, D, F, and H)** and the Cas9 variants **(J, L, and N)**. A greater separation between target and mismatch curves indicates higher specificity. All Cas RNP digestions were at 30nM to 3nM nucleosome library.

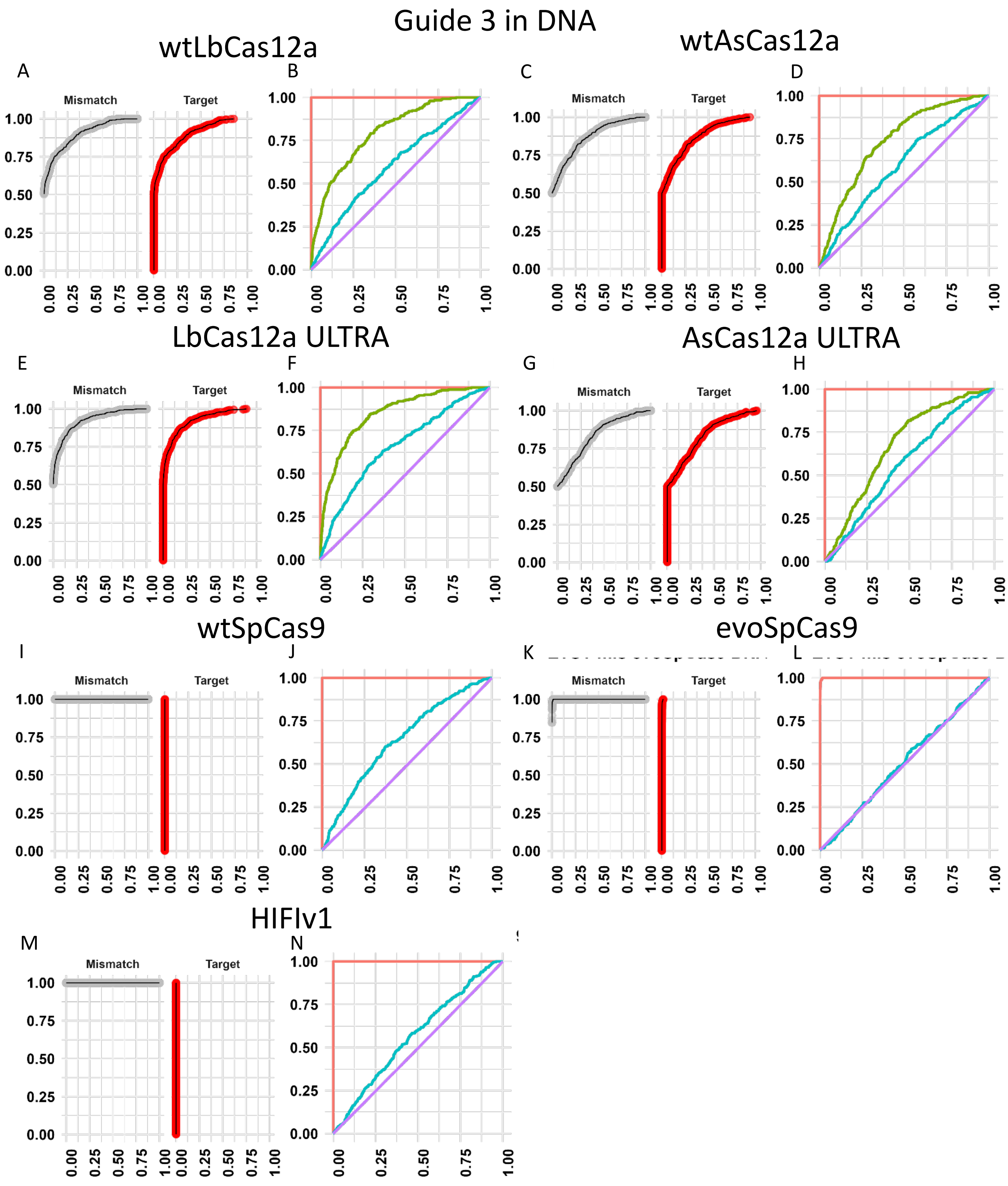

Supplemental Figure 21. Assessing the specificity and sensitivity of each Cas editor.
The 7,500 sequence library was used to evaluate the performance of each Cas editor. Sequences were ranked by cleavage efficiency, and true positive rate (TPR) versus false positive rate (FPR) was calculated to generate receiver operating characteristic (ROC) curves for **(A)** wtLbCas12a, **(C)** wtAsCas12a, **(E)** LbCas12a ULTRA, **(G)** AsCas12a ULTRA, **(I)** wtSpCas9, **(K)** evoSpCas9, and **(M)** HIFIv1. Each ROC curve is subdivided into targets within ± 60bp of the dyad, targets outside ± 60bp, and mistarget sequences. The cumulative frequency for the target sequences and varying amounts of mismatches (1-2, 3-4, and 5+) for the Cas12a variants **(B, D, F, and H)** and the Cas9 variants **(J, L, and N)**. A greater separation between target and mismatch curves indicates higher specificity. All Cas RNP digestions were at 30nM to 3nM nucleosome library.

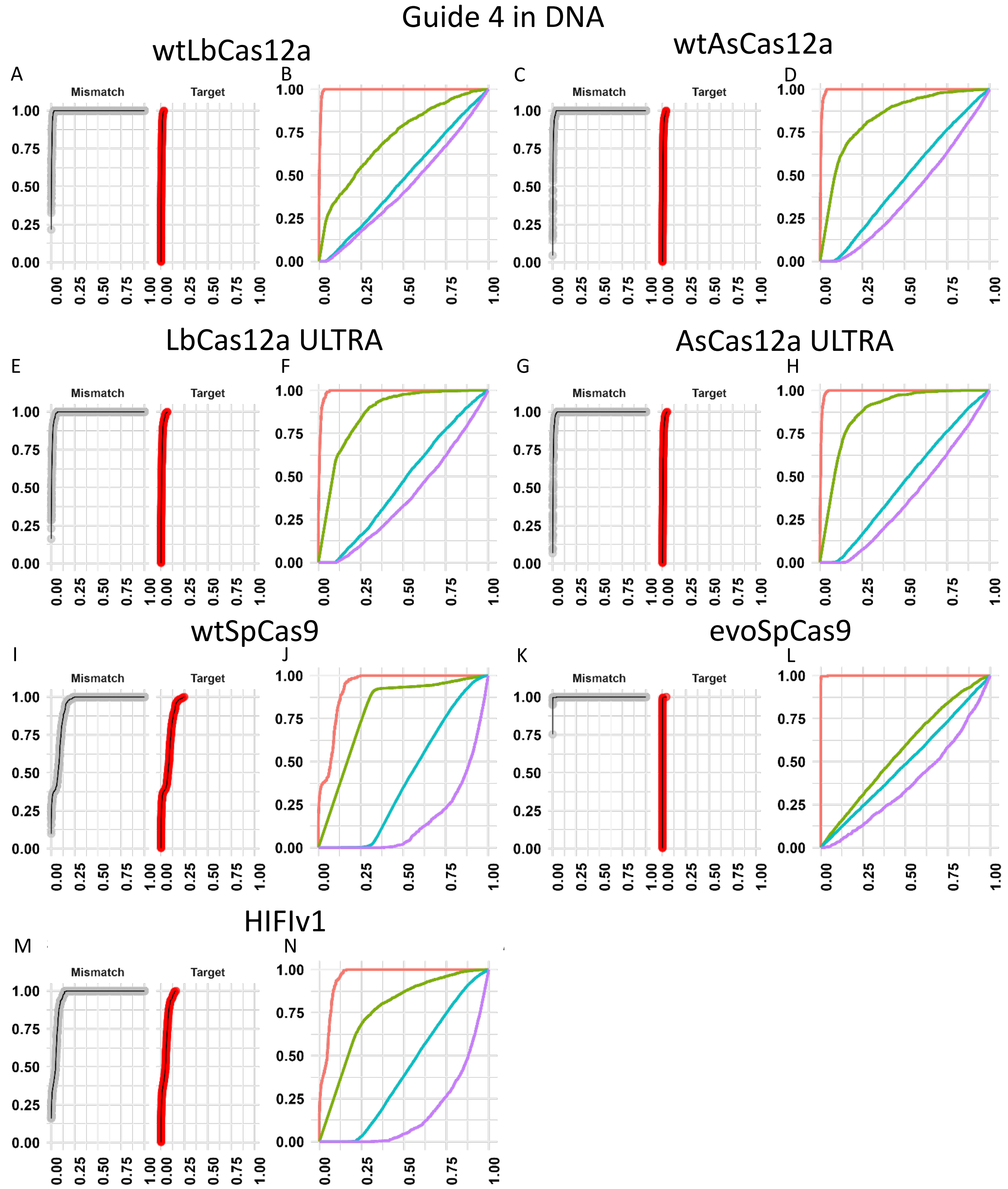

Supplemental Figure 22. Assessing the specificity and sensitivity of each Cas editor.
The 7,500 sequence library was used to evaluate the performance of each Cas editor. Sequences were ranked by cleavage efficiency, and true positive rate (TPR) versus false positive rate (FPR) was calculated to generate receiver operating characteristic (ROC) curves for **(A)** wtLbCas12a, **(C)** wtAsCas12a, **(E)** LbCas12a ULTRA, **(G)** AsCas12a ULTRA, **(I)** wtSpCas9, **(K)** evoSpCas9, and **(M)** HIFIv1. Each ROC curve is subdivided into targets within ± 60bp of the dyad, targets outside ± 60bp, and mistarget sequences. The cumulative frequency for the target sequences and varying amounts of mismatches (1-2, 3-4, and 5+) for the Cas12a variants **(B, D, F, and H)** and the Cas9 variants **(J, L, and N)**. A greater separation between target and mismatch curves indicates higher specificity. All Cas RNP digestions were at 30nM to 3nM nucleosome library.

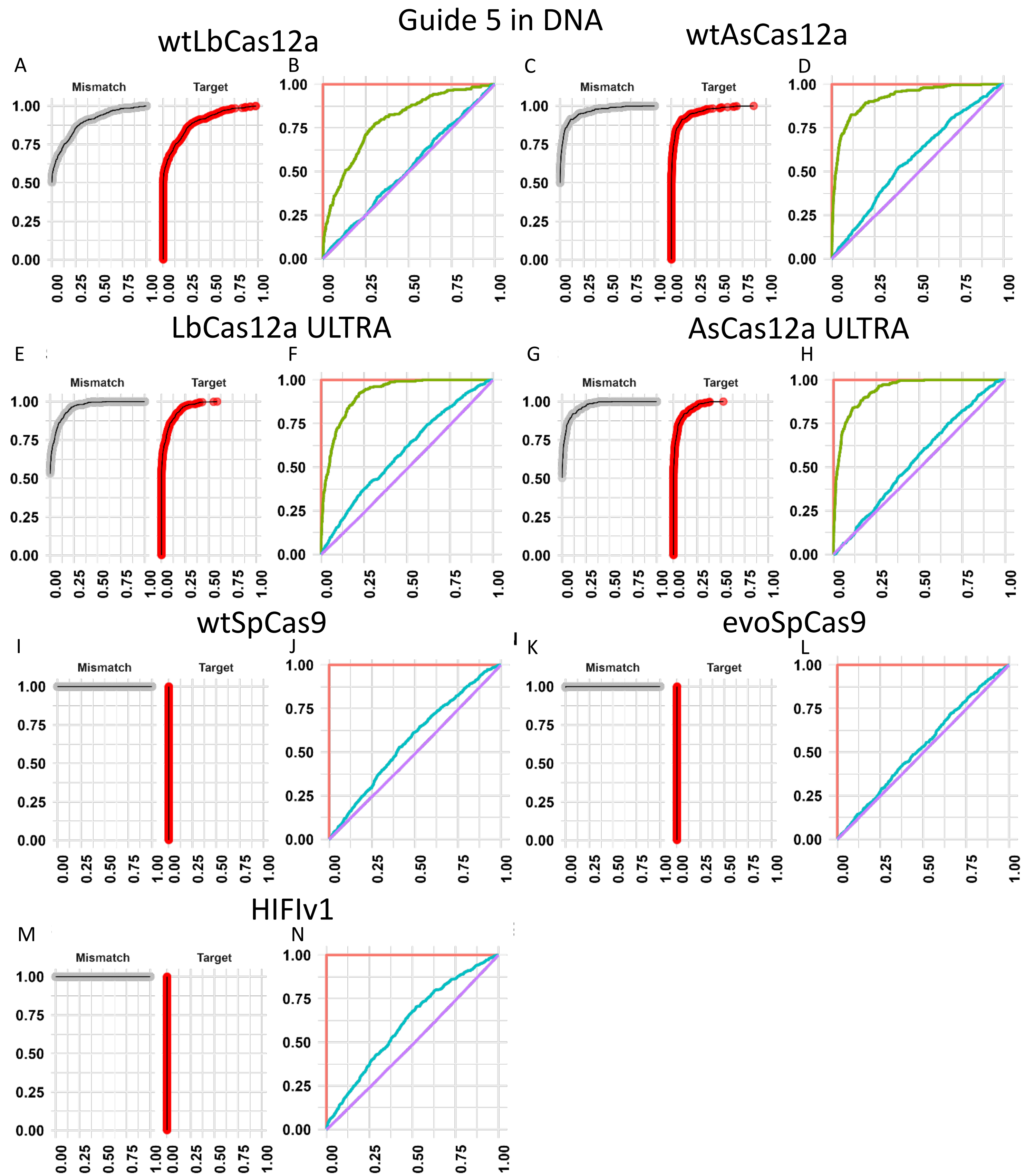

Supplemental Figure 23. Assessing the specificity and sensitivity of each Cas editor.
The 7,500 sequence library was used to evaluate the performance of each Cas editor. Sequences were ranked by cleavage efficiency, and true positive rate (TPR) versus false positive rate (FPR) was calculated to generate receiver operating characteristic (ROC) curves for **(A)** wtLbCas12a, **(C)** wtAsCas12a, **(E)** LbCas12a ULTRA, **(G)** AsCas12a ULTRA, **(I)** wtSpCas9, **(K)** evoSpCas9, and **(M)** HIFIv1. Each ROC curve is subdivided into targets within ± 60bp of the dyad, targets outside ± 60bp, and mistarget sequences. The cumulative frequency for the target sequences and varying amounts of mismatches (1-2, 3-4, and 5+) for the Cas12a variants **(B, D, F, and H)** and the Cas9 variants **(J, L, and N)**. A greater separation between target and mismatch curves indicates higher specificity. All Cas RNP digestions were at 30nM to 3nM nucleosome library.
