# Supplementary material for: Evaluating high-fidelity CRISPR-Cas nucleases in nucleosomal contexts using a quantitative framework
Source: Front Genome Ed. 2026 Aug 14;8:1759382. doi: 10.3389/fgeed.2026.1759382 (PMC13522198; doi:10.3389/fgeed.2026.1759382)
Supplement: Supplementary file 2 [file DataSheet2.pdf]

Supplemental Table 1. Dyad proximity increases the Cas nuclease concentration required for target cleavage across nucleosome positioning sequences.

NPS	Editor	rho	q-value
5S	ultLbCas12a	-0.83	5.87E-48
5S	wtSpCas9	-0.82	9.43E-46
5S	HIFIv1	-0.80	8.51E-44
5S	wtLbCas12a	-0.80	7.35E-40
5S	HIFIv2	-0.80	1.55E-42
5S	ultAsCas12a	-0.74	1.28E-33
5S	wtAsCas12a	-0.74	2.06E-32
5S	evoSpCas9	-0.50	1.07E-10
MMTV-NUCA	wtSpCas9	-0.91	2.91E-62
MMTV-NUCA	wtLbCas12a	-0.90	4.59E-57
MMTV-NUCA	ultLbCas12a	-0.88	1.06E-55
MMTV-NUCA	wtAsCas12a	-0.88	2.06E-50
MMTV-NUCA	HIFIv2	-0.85	2.91E-46
MMTV-NUCA	ultAsCas12a	-0.80	3.01E-38
MMTV-NUCA	HIFIv1	-0.71	1.07E-26
MMTV-NUCA	evoSpCas9	-0.28	2.94E-03
WIDOM-601-MOD	ultLbCas12a	-0.83	6.08E-44
WIDOM-601-MOD	wtAsCas12a	-0.82	4.75E-40
WIDOM-601-MOD	wtLbCas12a	-0.77	4.05E-26
WIDOM-601-MOD	HIFIv1	-0.73	2.11E-26
WIDOM-601-MOD	ultAsCas12a	-0.66	1.82E-21
WIDOM-601-MOD	wtSpCas9	-0.65	2.80E-22
WIDOM-601-MOD	HIFIv2	-0.61	5.70E-19
WIDOM-601-MOD	evoSpCas9	-0.59	1.36E-15

Spearman rank correlations were calculated for each Cas editor within each NPS to assess the relationship between KC values and distance from the nucleosome dyad. Benjamini–Hochberg-adjusted q-values are used for significance testing.

Tile sequences

ID	Sequence	PAM
**Sequence 1**	GCAAGAGGAAGTGAACGTAG	TGGAAA
Sequence 2	GCAAGATGAAGTGAACGTAG	CGGAAT
Sequence 3	GCAAGAGTAAGTGAACGTAG	CGGAAT
Sequence 4	GCAAGAGGCAGTGAACGTAG	TGGAAA
Sequence 5	GCAAGAGGAAGCGAACGTAG	TGGAAA
Sequence 6	GCAAGATTAAGTGAACGTAG	CGGAAT
Sequence 7	GCAAGATGCAGTGAACGTAG	CGGAAT
Sequence 8	GCAAGATGAAGCGAACGTAG	CGGAAT
Sequence 9	GCAAGAGTCAGTGAACGTAG	TGGAAA
Sequence 10	GCAAGAGTAAGCGAACGTAG	TGGAAA
Sequence 11	GCAAGAGGCAGCGAACGTAG	TGGAAA
Sequence 12	GCAAGATTCAGTGAACGTAG	CGGAAT
Sequence 13	GCAAGATGCAGCGAACGTAG	TGGAAA
Sequence 14	GCAAGAGGAAGTGAACGTAG	CGGAAT
Sequence 15	TCAAGAGGAAGTGAACGTAG	TGGAGA
Sequence 16	GCAAGAGGAAGTGAACGTAG	CGTAAT
**Sequence 17**	GCAAGAGGAAGTAAACGTAG	TGGAAA
Sequence 18	GTAAGATGAAGTAAACTTAA	CGGAAT
Sequence 19	GCAACCGGAAGTGAACGTAG	TGGAAA
Sequence 20	GCGACCGGACGTGCACGGAG	CGGAAT
Sequence 21	GCAACCGGAAATAAACGTAG	CGGAAT
Sequence 22	GCAAGCGGAAGTGAACGTAG	TGGAAA
**Sequence 23**	GTAAGATGAAGTAAACTTAA	TGGAAA
**Sequence 24**	GCGACCGGACGTGCACGGAG	TGGAAA
**Sequence 25**	GCACCCGGATGTAAACGTAG	TGGAAA
Sequence 26	GCAACAGGAAGTGAACGTAG	CGGAAT
Sequence 27	GAACCCGGAAGTAAACGTAG	CGGAAT
Sequence 28	TACCTCTAGCTATCCATGCT	TGGAAA
Sequence 29	TACCTCTAGCTATCCATGCT	CGGAAT

Supplemental Table 2. A nucleosome library of heterogeneous tiles provides an assessment of tolerance to many different potential off-Sequences. The various sequences all contain a variety of similarities, providing different on-target sequences to be compared with various off-targets with varying mismatch locations and number of base mispairings. Guide RNAs were designed to complement the sequences bolded and underlined in the table.

Nucleosome Positioning Sequences

WIDOM-601-MOD	GATGGACCCTATACGCGGCAAAGCATGATTCTCGCCCTGGAGAATCCCGGTGCCGAGGCCG<br>CTCAATTGGTCGTAGACAGCTCTAGCACCGCTTAAACGCACGTACGCGCTGTCCCCCGCGT<br>TTTAACCGCCAAGGGGATTACTCCCTAGTCTCCAGGCGCGTGTCAGATATATACATCCTGT<br>GAGGTTATTGAACAGCGACTTTACCGGTGCCAGTCGGATAGTGTTCC
5S	GATGGACCCTATACGCGGCGAGGAATTCCAACGAATAACTTCCAGGGATTTATAAGCCGAT<br>GACGTCATAACATCCCTGACCCTTTAAATAGCTTAACTTTCATCAAGCAAGAGCCTACGAC<br>CATACCATGCTGAATATACCGGTTCTCGTCCGATCACCGAAGTCAAGCAGCATAGGGCTCG<br>GTTAGTACTTGGATGGGAGACCGCCTGTGCCAGTCGGATAGTGTTCC
MMTV-NUCA	GATGGACCCTATACGCGGCAGAGTGCTGATTTTTTGAGTAAACTTGCAACAGTCCTAACAT<br>TCACCTCTTGTGTGTTTGTGTCTGTTCGCCATCCCGTCTCCGCTCGTCACTTATCCTTCAC<br>TTTCCAGAGGGTCCCCCCGCAGACCCCGGCGACCCTCAGGTCGGCCGACTGCGGCACAGTT<br>TTTTGCTCCTTTTTCTAGATGTAATTTTGCCAGTCGGATAGTGTTCC
5S-Lock	GATGGACCCTATACGCGGCAAAAAAAAAAAACGAATAACTTCCAGGGATTTATAAGCCGATGACG<br>TCATAACATCCCTGACCCTTTAAATAGCTTAACTTTCATCAAGCAAGAGCCTACGACCATACCATGC<br>TGAATATACCGGTTCTCGTCCGATCACCGAAGTCAAGCAGCATAGAAAAAAAAAAGTACTTGGAT<br>GGGAGACCGCCTGTGCCAGTCGGATAGTGTTCC
MMTV-NucA-Lock	GATGGACCCTATACGCGGCTATATTGAGTGTAAAAAAAAAAACTTGCAACAGTCCTAACATTCACC<br>TCTTGTGTGTTTGTGTCTGTTCGCCATCCCGTCTCCGCTCGTCACTTATCCTTCACTTTCCAGAGGGT<br>CCCCCCGCAGACCCCGGCGACCCTCAGGTCGGCCGACTGCGGCACAGTTTTTTGAAAAAAAAAAT<br>AGATGTAATTTTGCCAGTCGGATAGTGTTCC

Supplemental Table 3. Nucleosome Positioning Sequences.

Roc and PRC tables

Area Under the Receiver Operational Curve

Naked DNA	
Cas Editors	Average
evoSpCas9 DNA	0.991 ± 0.003
HIFIv1SpCas9 DNA	0.957 ± 0.025
wtSpCas9 DNA	0.944 ± 0.03
ultLbCas12a DNA	0.915 ± 0.047
wtLbCas12a DNA	0.886 ± 0.056
wtAsCas12a DNA	0.885 ± 0.064
ultAsCas12a DNA	0.879 ± 0.067

Nucleosomal DNA	
Cas Editors	Average
HIFIv1SpCas9 Nuc	0.815 ± 0.033
ultLbCas12a Nuc	0.811 ± 0.044
wtSpCas9 Nuc	0.775 ± 0.043
evoSpCas9 Nuc	0.772 ± 0.026
wtLbCas12a Nuc	0.758 ± 0.052
ultAsCas12a Nuc	0.744 ± 0.047
wtAsCas12a Nuc	0.731 ± 0.037

Area Under the Precision-Recall Curve

Naked DNA	
Cas Editors	Average
evoSpCas9 DNA	0.931 ± 0.06
ultLbCas12a DNA	0.736 ± 0.101
HIFIv1SpCas9 DNA	0.714 ± 0.137
wtLbCas12a DNA	0.708 ± 0.113
wtAsCas12a DNA	0.688 ± 0.134
ultAsCas12a DNA	0.675 ± 0.133
wtSpCas9 DNA	0.65 ± 0.143

Nucleosomal DNA	
Cas Editors	Average
evoSpCas9 Nuc	0.348 ± 0.049
HIFIv1SpCas9 Nuc	0.41 ± 0.088
ultAsCas12a Nuc	0.303 ± 0.064
ultLbCas12a Nuc	0.398 ± 0.067
wtAsCas12a Nuc	0.299 ± 0.058
wtLbCas12a Nuc	0.347 ± 0.077
wtSpCas9 Nuc	0.346 ± 0.082

Supplemental Table 4. The AUROC and AUPRC values for each nuclease within both naked DNA and nucleosomes. The scores go from 0 to 1, and values closet to 1 show better performance in both the ROC and the PRC curves. All AUROC and AUPRC is the average and standard error of the mean from 5 replicates.

Supplemental Table 5. The fold changes in Cas editor cleavage between the various NPS by distance from dyad. Fold change and statistical comparisons were performed at the nucleotide level using two-tailed Welch's t-tests on Cas editor cleavage values from 120nM digests. The p-values were adjusted for multiple testing using the Benjamini–Hochberg false discovery rate (FDR) procedure. All comparisons are between Cas editors with 3 replicates.

Distance from dyad	wtSpCas9 5S vs Widom-601		wtSpCas9 MMTV vs Widom-601		ultraLbCas12a 5S vs Widom-601		ultraLbCas12a MMTV vs Widom-601	
	Fold Change	q-value	Fold Change	q-value	Fold Change	q-value	Fold Change	q-value
-79	0.454	0.422	0.013	0.057	3.950	0.086	0.002	0.038
-78	0.952	0.981	0.019	0.062	3.464	0.114	0.004	0.056
-77	0.505	0.522	0.032	0.082	2.446	0.124	0.003	0.038
-76	0.139	0.154	0.022	0.057	1.044	0.099	0.000	0.032
-75	2.578	0.660	0.136	0.274	0.005	0.847	0.002	0.052
-74	0.319	0.469	0.084	0.062	0.021	0.754	0.004	0.049
-73	0.796	0.705	0.974	0.447	2.428	0.083	0.001	0.032
-72	2.961	0.099	0.511	0.212	24.224	0.102	0.062	0.060
-71	0.831	0.766	0.296	0.173	0.246	0.861	0.029	0.057
-70	2.271	0.469	0.382	0.326	0.001	0.852	0.008	0.043
-69	2.371	0.558	0.111	0.356	1.808	0.138	0.002	0.034
-68	1.391	0.735	0.118	0.067	3.765	0.048	0.010	0.041
-67	3.298	0.242	0.669	0.459	0.671	0.474	0.523	0.596
-66	2.017	0.474	0.406	0.809	3.176	0.626	0.700	0.785
-65	4.395	0.150	1.599	0.842	3.048	0.147	0.613	0.333
-64	5.337	0.099	1.660	0.636	0.355	0.046	1.826	0.436
-63	6.594	0.154	1.287	0.770	0.967	0.136	2.336	0.272
-62	6.914	0.099	2.172	0.812	0.147	0.046	2.017	0.530
-61	7.394	0.099	2.073	0.285	0.117	0.037	0.662	0.334
-60	4.977	0.132	1.455	0.612	27.444	0.048	2.806	0.182
-59	10.463	0.099	0.652	0.459	0.016	0.038	2.453	0.154
-58	15.006	0.131	3.915	0.649	4.638	0.086	1.975	0.342
-57	11.647	0.147	1.974	0.119	7.969	0.101	1.482	0.530
-56	4.224	0.150	2.257	0.446	1.815	0.238	1.635	0.342
-55	3.159	0.168	0.814	0.761	0.756	0.046	3.316	0.030
-54	6.602	0.075	2.472	0.334	2.843	0.048	2.695	0.067
-53	1.571	0.075	1.093	0.511	0.975	0.124	1.925	0.151
-52	1.297	0.735	0.939	0.975	17.150	0.048	1.386	0.442
-51	1.782	0.503	2.850	0.743	7.405	0.111	1.944	0.580
-50	5.398	0.168	7.179	0.119	4.643	0.086	2.509	0.165
-49	4.160	0.099	3.469	0.147	0.011	0.046	0.577	0.142
-48	3.459	0.168	2.498	0.337	3.320	0.119	2.127	0.114
-47	2.160	0.469	2.222	0.296	3.816	0.119	4.715	0.042
-46	3.637	0.380	0.780	0.518	26.816	0.086	2.813	0.103
-45	4.257	0.195	2.075	0.595	6.757	0.114	3.157	0.121
-44	2.143	0.157	2.716	0.514	7.632	0.086	2.672	0.174
-43	3.047	0.443	1.036	0.557	2.040	0.190	1.385	0.427
-42	3.629	0.107	4.828	0.514	2.167	0.111	1.212	0.766
-41	2.923	0.278	0.322	0.157	1.150	0.155	1.063	0.952
-40	2.429	0.503	0.535	0.175	282.503	0.048	1.226	0.768
-39	5.645	0.238	0.815	0.628	1.550	0.231	0.410	0.196
-38	10.215	0.099	2.071	0.830	2.042	0.154	0.353	0.244

-37	1.672	0.755	1.311	0.757	7.925	0.119	0.749	0.718
-36	6.646	0.154	1.224	0.738	16.911	0.124	1.492	0.640
-35	3.047	0.177	0.359	0.832	5.477	0.326	1.500	0.741
-34	3.765	0.268	0.468	0.212	2.973	0.155	1.876	0.442
-33	3.418	0.284	1.180	0.898	1.941	0.683	0.314	0.192
-32	1.614	0.735	0.323	0.459	1.212	0.046	1.893	0.319
-31	0.871	0.885	0.605	0.560	24.337	0.083	2.899	0.049
-30	10.982	0.099	2.561	0.212	0.031	0.038	2.159	0.114
-29	2.024	0.422	1.596	0.709	0.772	0.125	0.593	0.535
-28	4.673	0.146	1.333	0.700	9.299	0.080	1.020	0.969
-27	4.629	0.168	0.455	0.612	1.329	0.319	1.568	0.328
-26	4.553	0.146	1.154	0.274	5.847	0.062	2.134	0.342
-25	5.523	0.132	0.916	0.649	0.001	0.985	0.350	0.478
-24	7.474	0.154	0.349	0.511	47.218	0.111	2.473	0.296
-23	1.570	0.650	0.913	0.212	2.252	0.166	1.868	0.109
-22	3.072	0.147	4.760	0.285	2.223	0.119	1.603	0.449
-21	1.480	0.705	0.726	0.459	8.568	0.080	3.171	0.171
-20	0.963	0.981	1.609	0.898	4.614	0.119	2.947	0.113
-19	3.403	0.443	0.674	0.770	6.663	0.048	2.216	0.165
-18	1.166	0.806	0.578	0.251	9.226	0.164	2.249	0.361
-17	1.111	0.832	0.757	0.741	0.983	0.320	0.956	0.940
-16	4.532	0.150	0.884	0.700	0.034	0.715	1.109	0.718
-15	1.020	0.982	1.068	0.459	0.001	0.773	1.435	0.715
-14	2.500	0.380	2.730	0.286	10.276	0.052	1.422	0.328
-13	1.687	0.512	1.442	0.471	9.743	0.086	1.346	0.328
-12	1.253	0.735	1.051	0.741	4.468	0.160	3.151	0.041
-11	3.153	0.316	1.450	0.738	2.047	0.048	3.287	0.103
-10	2.328	0.168	1.773	0.212	11.576	0.124	2.117	0.144
-9	2.876	0.445	0.746	0.738	4.639	0.280	1.823	0.442
-8	4.966	0.129	0.875	0.512	0.610	0.142	4.319	0.079
-7	1.719	0.566	1.237	0.738	0.516	0.119	3.438	0.120
-6	2.683	0.443	1.546	0.337	7.927	0.054	4.712	0.132
-5	1.113	0.805	1.132	0.535	3.754	0.352	1.656	0.446
-4	1.667	0.540	0.825	0.514	4.258	0.119	2.514	0.093
-3	1.603	0.614	1.170	0.598	3.735	0.060	2.002	0.152
-2	0.633	0.705	1.208	0.832	5.042	0.048	4.092	0.150
-1	2.298	0.154	1.402	0.285	3.411	0.062	3.045	0.141
0	3.795	0.099	0.894	0.781	4.185	0.154	3.417	0.194
1	2.283	0.397	0.621	0.212	0.006	0.037	5.939	0.056
2	1.036	0.927	1.337	0.570	4.572	0.314	2.881	0.146
3	1.993	0.503	2.913	0.485	0.030	0.037	7.101	0.104
4	1.245	0.755	0.898	0.743	0.138	0.973	4.686	0.109
5	1.077	0.832	1.342	0.830	0.001	0.773	1.693	0.417
6	2.432	0.368	2.652	0.382	0.007	0.983	6.424	0.043
7	0.588	0.445	0.507	0.585	1.243	0.439	7.850	0.067
8	1.136	0.735	0.846	0.905	0.818	0.475	2.637	0.295
9	1.718	0.653	1.332	0.535	3.534	0.178	2.490	0.032
10	1.661	0.662	0.744	0.649	0.700	0.245	2.837	0.079
11	1.884	0.578	0.747	0.740	2.381	0.083	7.426	0.069
12	1.051	0.954	0.600	0.212	4.434	0.119	8.766	0.049

13	4.195	0.146	0.986	0.721	9.085	0.149	23.414	0.022
14	0.688	0.578	1.903	0.754	0.001	0.861	26.843	0.049
15	1.674	0.548	4.302	0.216	0.854	0.201	23.563	0.049
16	2.469	0.290	4.624	0.213	4.676	0.124	8.675	0.049
17	6.543	0.099	3.470	0.285	0.578	0.086	27.275	0.057
18	4.125	0.115	6.171	0.135	0.000	0.992	12.349	0.049
19	2.701	0.290	9.913	0.535	0.675	0.453	11.502	0.057
20	10.469	0.085	3.040	0.119	3.079	0.272	6.454	0.058
21	5.455	0.278	4.336	0.459	4.573	0.102	2.492	0.050
22	2.168	0.114	2.050	0.456	0.587	0.474	12.556	0.078
23	2.353	0.558	2.017	0.577	0.012	0.889	8.285	0.150
24	5.522	0.146	8.206	0.147	0.000	0.927	13.494	0.057
25	1.539	0.653	14.009	0.181	0.098	0.710	3.247	0.165
26	1.808	0.558	49.318	0.093	33.777	0.086	8.680	0.032
27	2.145	0.278	8.482	0.057	0.020	0.717	19.154	0.064
28	0.542	0.548	3.407	0.279	0.877	0.477	11.858	0.065
29	4.169	0.099	10.053	0.041	7.808	0.676	4.412	0.036
30	2.502	0.115	13.142	0.236	3.488	0.048	12.297	0.028
31	1.158	0.721	20.507	0.171	2.196	0.174	13.758	0.032
32	3.663	0.278	13.776	0.057	1.214	0.111	18.172	0.038
33	1.193	0.788	8.878	0.179	0.012	0.824	14.721	0.040
34	1.574	0.503	8.334	0.147	6.919	0.554	7.190	0.171
35	2.176	0.330	7.803	0.057	4.089	0.048	60.548	0.022
36	1.338	0.766	6.043	0.115	1.419	0.603	9.185	0.038
37	5.432	0.075	22.113	0.057	0.000	0.991	9.922	0.057
38	1.570	0.548	15.192	0.175	34.926	0.048	27.441	0.091
39	3.842	0.154	41.428	0.057	4.355	0.268	49.936	0.006
40	0.675	0.284	12.959	0.015	2.286	0.554	10.854	0.120
41	2.974	0.379	20.472	0.023	0.006	0.883	12.530	0.038
42	1.969	0.379	64.955	0.135	0.009	0.756	21.832	0.103
43	3.356	0.292	12.352	0.062	0.060	0.724	224.855	0.000
44	0.532	0.523	9.171	0.285	0.056	0.623	12.533	0.057
45	0.989	0.991	13.814	0.151	0.047	0.861	6.942	0.058
46	0.763	0.760	26.689	0.057	0.011	0.887	6.076	0.150
47	4.416	0.254	36.094	0.076	0.727	0.299	6.910	0.074
48	3.789	0.099	21.962	0.093	1.352	0.205	10.602	0.064
49	3.680	0.101	25.987	0.057	0.004	0.807	5.915	0.038
50	4.423	0.154	12.356	0.062	1.818	0.065	0.311	0.270
51	4.864	0.163	20.211	0.296	3.188	0.216	9.702	0.070
52	10.450	0.099	44.226	0.212	2.744	0.062	5.679	0.146
53	2.337	0.284	39.511	0.173	0.925	0.201	2.086	0.436
54	0.997	0.994	13.245	0.067	1.417	0.111	0.214	0.154
55	0.515	0.391	5.378	0.407	20.089	0.094	0.015	0.067
56	1.441	0.766	16.400	0.195	2.402	0.046	0.006	0.030
57	2.263	0.474	13.040	0.159	3.741	0.062	0.014	0.032
58	1.092	0.806	2.649	0.212	5.015	0.119	0.006	0.084
59	1.307	0.735	1.261	0.459	6.865	0.102	0.004	0.056
60	12.484	0.150	2.710	0.612	59.311	0.048	0.003	0.049
61	0.723	0.805	0.429	0.057	0.020	0.037	0.001	0.028
62	0.127	0.132	0.130	0.062	2.940	0.086	0.001	0.065

63	0.060	0.278	0.023	0.212	0.665	0.041	0.002	0.049
64	0.055	0.099	0.132	0.057	24.305	0.052	0.000	0.032
65	0.011	0.085	0.021	0.057	0.721	0.041	0.000	0.022
66	0.040	0.278	0.011	0.242	67.423	0.052	0.001	0.043
67	0.062	0.101	0.037	0.057	6.820	0.048	0.003	0.028
68	0.073	0.150	0.040	0.157	0.601	0.352	0.032	0.077
69	0.064	0.154	0.120	0.147	7.552	0.111	0.002	0.068
70	0.101	0.168	0.030	0.459	4.561	0.119	0.001	0.058
71	0.071	0.075	0.067	0.057	12.072	0.124	0.007	0.098
72	0.121	0.347	0.026	0.212	16.376	0.086	0.001	0.089
